# Defect engineering of layered double hydroxide nanosheets as inorganic photosensitizers for NIR-III photodynamic cancer therapy

**DOI:** 10.1038/s41467-022-31106-9

**Published:** 2022-06-13

**Authors:** Weicheng Shen, Tingting Hu, Xueyan Liu, Jiajia Zha, Fanqi Meng, Zhikang Wu, Zhuolin Cui, Yu Yang, Hai Li, Qinghua Zhang, Lin Gu, Ruizheng Liang, Chaoliang Tan

**Affiliations:** 1 State Key Laboratory of Chemical Resource Engineering, Beijing Advanced Innovation Center for Soft Matter Science and Engineering, Beijing University of Chemical Technology, Beijing, 100029 China; 2 Department of Electrical Engineering, City University of Hong Kong, 83 Tat Chee Avenue, Kowloon, Hong Kong; 3 Beijing National Laboratory for Condensed Matter Physics, Institute of Physics, Chinese Academy of Sciences, Beijing, 100190 China; 4 Institute of Advanced Materials (IAM) and Key Laboratory of Flexible Electronics (KLoFE), Nanjing Tech University (NanjingTech), 30 South Puzhu Road, Nanjing, 211816 China; 5 School of Physical Sciences, University of Chinese Academy of Sciences, Beijing, 100049 China; 6 Center of Super-Diamond and Advanced Films (COSDAF), City University of Hong Kong, Kowloon, Hong Kong; 7 Shenzhen Research Institute, City University of Hong Kong, Shenzhen, 518057 China

**Keywords:** Cancer therapy, Bioinorganic chemistry, Nanoparticles

## Abstract

Although two-dimensional (2D) layered double hydroxides (LDHs) have been widely used as efficient nanoagents for biological diagnosis and treatment, they have been found to be inert as photosensitizers (PSs) for photodynamic therapy (PDT). Herein, we report the defect engineering of ultrathin 2D CoMo-LDH and NiMo-LDH nanosheets as highly active inorganic PSs for PDT in the third near-infrared (NIR-III) window. Hydrothermal-synthesized 2D CoMo-LDH and NiMo-LDH nanosheets are etched via a simple acid treatment to obtain defect-rich CoMo-LDH and NiMo-LDH nanosheets. Importantly, the defect-rich CoMo-LDH nanosheets exhibit much higher activity (~97 times) for generation of reactive oxygen species than that of the pristine CoMo-LDH nanosheets under a NIR-III 1567 nm laser irradiation. Therefore, after modification with polyethylene glycol, the defect-rich CoMo-LDH nanosheets can be used as an efficient inorganic PS for PDT to efficiently induce cancer cells apoptosis in vitro and eradicate tumors in vivo under 1567 nm laser irradiation.

## Abstract (web summary)

Defect engineering of 2 dimensional layered double hydroxide sheets improves their photocatalytic activity. Here, the authors etch sheets in acid and show that the etched sheets generate substantially more reactive oxygen species that untreated sheets and the treated sheets can be used to kill cancer cells in vitro and in vivo.

## Introduction

Photodynamic therapy (PDT) has been proven to be one of the most promising ways for cancer treatment in recent years owing to its high spatiotemporal precision, limited side effects, and minimally invasive nature^1–4^. PDT generally involves the absorption of light by photosensitizers (PSs) to excite the generation of toxic reactive oxygen species (ROS), including hydroxyl radicals (·OH), superoxide radicals (·O_2_^−^) and singlet oxygen (^1^O_2_)^5–9^. Therefore, developing highly active PSs is essential to achieve excellent performance in PDT. In recent years, great effort has been devoted to the development of inorganic nanomaterials serving as PSs for near-infrared (NIR) PDT because of their superior advantages, such as excellent photostability and easy accumulation at tumor sites, as compared to widely explored organic PSs^10–18^. For example, inorganic nanomaterials, such as Au nanocages, graphene quantum dots, copper/manganese silicate nanosphere-coated lanthanide-doped nanoparticles, BiAgOS nanoparticles, Ti_3_C_2_/g-C_3_N_4_ heterostructure, Cu_2−*x*_S nanocrystals, W_18_O_49_ nanowires and Cs_*x*_WO_3_ nanorods, have been reported as efficient PSs for PDT in the first NIR window (NIR-I: 750–1000 nm)^19–26^. Moreover, few types of inorganic nanomaterials including Cu_2_(OH)PO_4_ quantum dots, gold nanoechinus and Ag-doped Au/CdSe_*x*_S_*y*_ have also been developed as PSs for PDT in NIR-II (1000–1350 nm) window^27–29^. These reported inorganic NIR-II PSs normally suffer from poor efficiency in ROS generation, thus significantly limiting their further applications in the elimination of cancer cells and tumors. It is worth pointing out that light in NIR-III window (1350–1870 nm) has larger maximum permissible exposure and longer penetration depth than the light in NIR-I and NIR-II windows, making it more promising as light source for PDT. However, it still remains a daunting challenge to develop inorganic PSs that can be excited by light in the NIR-III window.

Two-dimensional (2D) layered double hydroxide (LDH) nanosheets have been demonstrated to be promising nanoagents for biological diagnosis and treatment owing to their good biocompatibility, pH-sensitive biodegradability, highly tunable chemical composition and structure^30–36^. For example, ultrathin 2D MgAlGd-LDH nanosheets can be used as a carrier to load with chemical drugs for high-performance drug delivery thanks to its ultralarge surface area^37^. Moreover, ultrathin CoFe-LDH and CuFe-LDH nanosheets can trigger the Fenton or Fenton-like reactions to realize chemodynamic therapy (CDT) and thus induce tumor cells apoptosis by catalyzing the overexpression of H_2_O_2_ into toxic ·OH within tumor microenvironment^38,39^. Importantly, 2D LDH nanosheets have also been used as the host to intercalate with organic PSs in their interlayer spacings or load with organic PSs on their surface, enabling them as active PSs for PDT^40–43^. As a typical example, our group has reported that the isophthalic acid-intercalated ZnAl-LDH nanosheets exhibited a high ^1^O_2_ quantum yield when used as a PS for the NIR-I light-induced ROS generation, making it promising for PDT to eliminate cancer cells and tumors^41^. Despite the fact that 2D LDHs have been used as the appealing host to functionalize with organic PSs, it still remains a great challenge to develop 2D LDHs themselves as highly active inorganic PSs for NIR PDT.

**Fig. 1:**
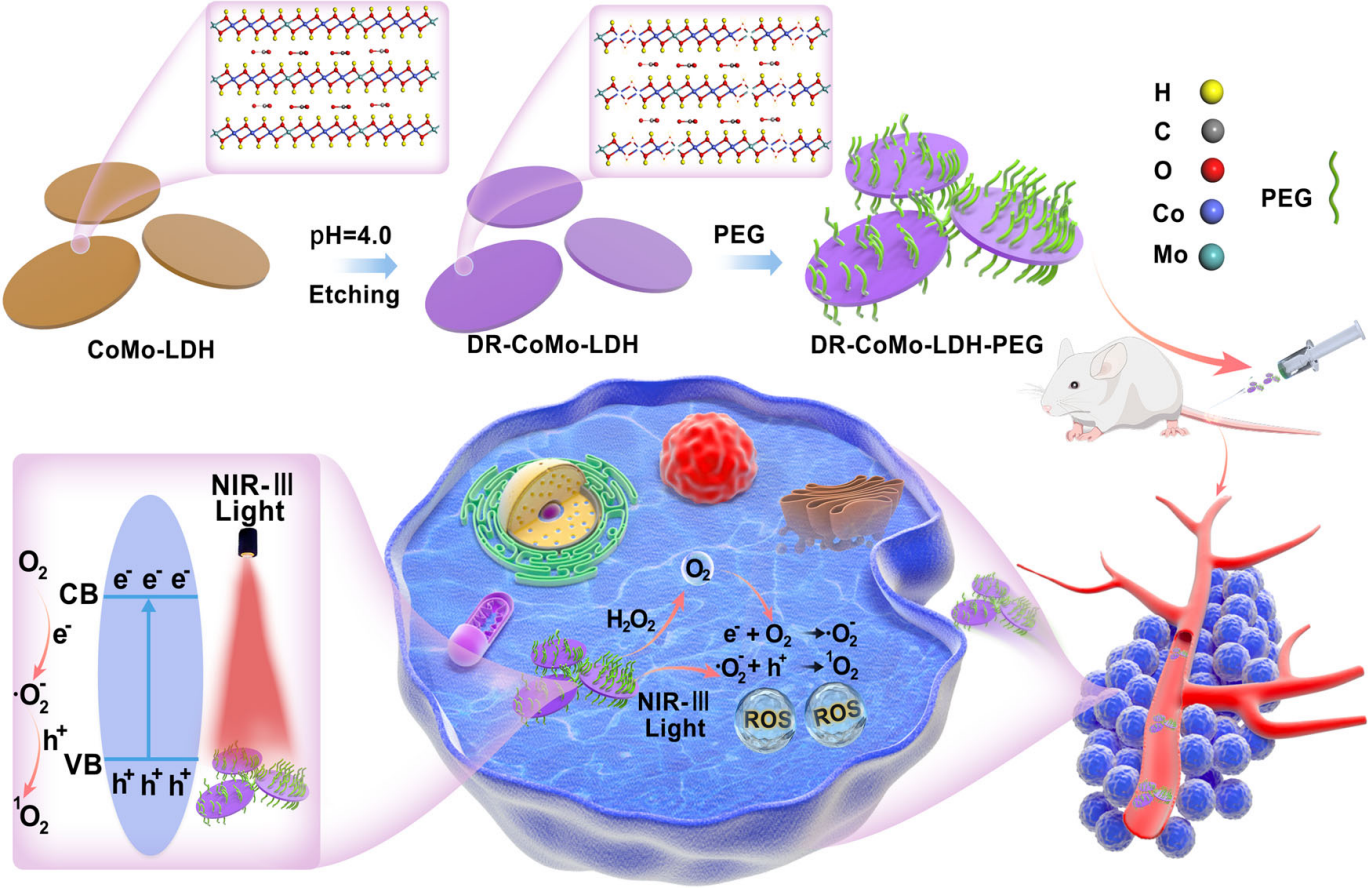
Schematic illustration of the defect engineering of CoMo-LDH nanosheets, surface modification with PEG and its application in NIR-III PDT. The as-prepared CoMo-LDH nanosheets are etched at pH 4.0 for 2 h, and then modified with PEG to obtain defect-rich DR-CoMo-LDH-PEG nanosheets. Under 1567 nm laser irradiation, DR-CoMo-LDH-PEG nanosheets can generate a large amount of ROS to eradicate tumors.

Defect engineering has been proven to be an effective strategy to significantly enhance the photocatalytic and electrocatalytic activities of 2D LDHs in recent years^44–46^, but how the defects will affect the PDT performance of 2D LDHs still remains unexplored. In this contribution, we report the defect engineering of ultrathin 2D CoMo-LDH and NiMo-LDH nanosheets via simple acid treatment as highly active inorganic PSs for NIR-III photodynamic cancer therapy (Fig. 1). Specifically, ultrathin 2D CoMo-LDH and NiMo-LDH nanosheets are synthesized by a hydrothermal method and then etched via a simple acid treatment to obtain defect-rich CoMo-LDH and NiMo-LDH nanosheets. Importantly, when used as PS, the defect-rich CoMo-LDH (denoted as DR-CoMo-LDH) nanosheets exhibit excellent activity towards the generation of ROS including ·O_2_^−^ and ^1^O_2_ under NIR-III 1567 nm laser irradiation (Fig. 1), whose activity is ~97 times of that the pristine CoMo-LDH nanosheets. Our results suggested that the 2D DR-CoMo-LDH nanosheets for highly efficient PDT could be attributed to the defect engineering-induced electronic structure changing. More importantly, both in vitro and in vivo experiments indicate that after polyethylene glycol (PEG) modification, the DR-CoMo-LDH nanosheets exhibit excellent performance in PDT for highly efficient cancer cell killing and tumor elimination under 1567 nm laser irradiation (Fig. 1). Our study reveals that defect engineering is a simple and effective approach to tune 2D LDHs as highly active PSs for NIR PDT.

## Results

### Synthesis and characterization of 2D LDH nanosheets

**Fig. 2:**
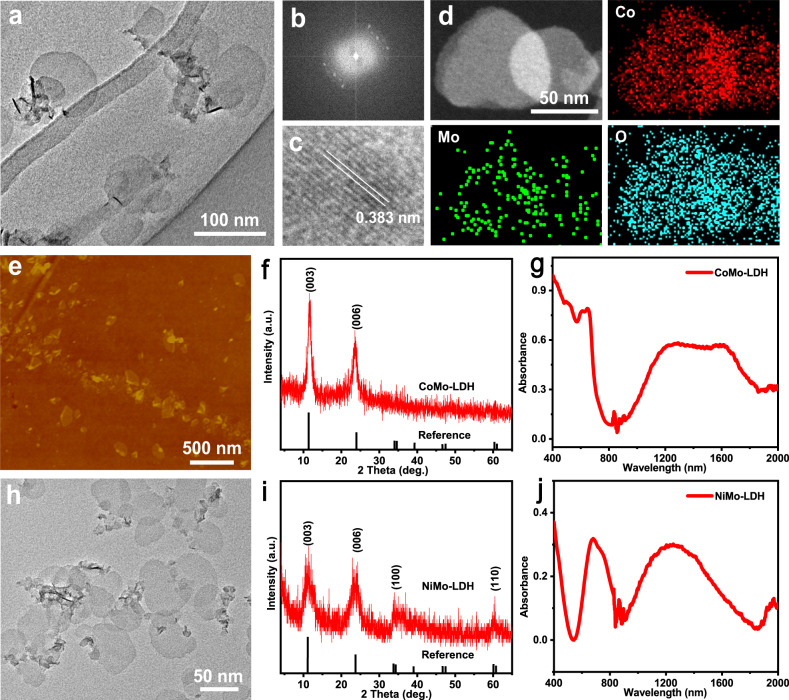
Structural characterizations of CoMo-LDH and NiMo-LDH nanosheets. **a** TEM image, **b** SAED pattern and **c** HRTEM image, **d** EDX mapping and **e** AFM image of the CoMo-LDH nanosheets. **f** XRD pattern of the CoMo-LDH nanosheets. **g** UV–vis–NIR diffuse reflection spectrum of the CoMo-LDH nanosheets. **h** TEM image, **i** XRD pattern of the NiMo-LDH nanosheets. **j** UV–vis–NIR diffuse reflection spectrum of the NiMo-LDH nanosheets. Each experiment was repeated three times with similar results in all these characterization figures. Source data are provided as a Source Data file.

Ultrathin 2D CoMo-LDH and NiMo-LDH nanosheets were synthesized by a simple low-temperature hydrothermal method (see details in Methods). As presented in Fig. 2a, the transmission electron microscopy (TEM) image clearly indicates the 2D nanosheet morphology with an ultrathin thickness of the synthesized CoMo-LDH sample. The size of the CoMo-LDH nanosheets is 50–80 nm. The selected area electron diffraction (SAED) pattern of a typical CoMo-LDH nanosheet gives a single set of diffract spots (Fig. 2b), indicating its good crystallinity. The high-resolution TEM (HRTEM) image of the CoMo-LDH nanosheets displays continuous lattice fringes and the measured lattice spacing is ~0.383 nm (Fig. 2c), which is corresponding to (006) planes of the CoMo-LDH crystal^47^. The energy-dispersive X-ray (EDX) elemental mapping reveals that Co, Mo, and O elements are homogeneously distributed across the whole nanosheets (Fig. 2d) and the elemental ratio of Co: Mo calculated from the EDX spectrum is around 5.9:1 (Supplementary Fig. 1). The thickness of the CoMo-LDH nanosheets is characterized by atomic force microscopy (AFM). The thickness of the CoMo-LDH nanosheets measured from its AFM height image is around 1.3–1.6 nm (Fig. 2e and Supplementary Fig. 2), indicating its ultrathin nature. Typical (003) and (006) diffraction peaks at 2*θ* = 11.54° and 24.38° in its X-ray diffraction (XRD) pattern can well match with the reference (Fig. 2f)^48^, further confirming its crystal structure. The absorption spectrum of the CoMo-LDH nanosheets was determined by an ultraviolet–visible–near-infrared (UV–vis–NIR) diffuse reflection spectrophotometer and it gives a broad and strong NIR absorption ranging from 900 to 1800 nm (Fig. 2g). Similarly, the NiMo-LDH nanosheets were also characterized by TEM, XRD, AFM and UV–vis–NIR diffuse reflection spectrum. The TEM image indicates that the NiMo-LDH nanosheets have a size of 40–80 nm with an ultrathin thickness (Fig. 2h). The XRD pattern of the NiMo-LDH nanosheets shows that all the characteristic diffraction peaks were well indexed by the standard reference (JCPDS No. 46-0605) (Fig. 2i)^49^, confirming its crystal structure. Its UV–vis–NIR diffuse reflection spectrum gives strong absorption in the NIR region (1000–1700 nm) (Fig. 2j). All the aforementioned characterization results have proven the successful preparation of ultrathin 2D CoMo-LDH and NiMo-LDH nanosheets.

### Catalytic activity of CoMo-LDH for ROS generation

**Fig. 3:**
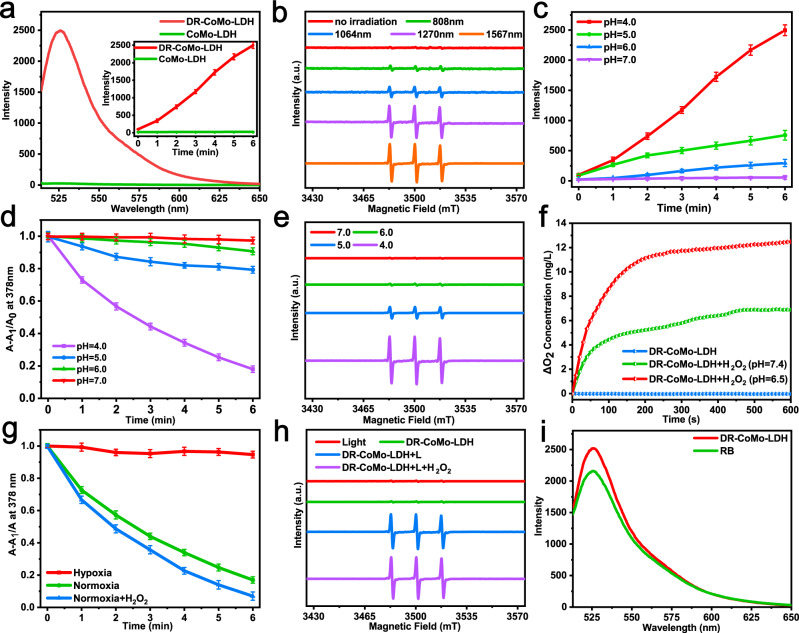
Identification of ^1^O_2_ generation. **a** The fluorescence intensity of SOSG in presence of the CoMo-LDH and DR-CoMo-LDH nanosheets in H_2_O under 1567 nm laser irradiation (0.5 W cm^−2^). Data are presented as mean values ± s.d. (*n* = 3). **b** ESR spectra of TEMP/^1^O_2_ for the DR-CoMo-LDH nanosheets in H_2_O under different-wavelength laser irradiation (0.5 W cm^−2^) for 6 min. **c** The fluorescence intensity of SOSG (in H_2_O) in presence of CoMo-LDH nanosheets etched at different pH for 2 h with 1567 nm laser irradiation (0.5 W cm^−2^). Data are presented as mean values ± s.d. (*n* = 3). **d** Normalized decay curves for the absorption of ABDA in H_2_O at 378 nm as a function of irradiation time (1567 nm, 0.5 W cm^−2^). Data are presented as mean values ± s.d. (*n* = 3). **e** ESR spectra of TEMP/^1^O_2_ (in H_2_O) for the CoMo-LDH nanosheets etched at different pH under 1567 nm laser irradiation (0.5 W cm^−2^, 6 min). **f** O_2_ concentration curve of the DR-CoMo-LDH nanosheets without H_2_O_2_ (blue) and with H_2_O_2_ in PBS solution at pH 7.4 (green) and 6.5 (red) for 10 min. **g** Normalized absorbance of ABDA (in H_2_O) in the presence of the DR-CoMo-LDH nanosheets under different environments. Data are presented as mean values ± s.d. (*n* = 3). **h** ESR spectra of TEMP/^1^O_2_ (in H_2_O) for the DR-CoMo-LDH nanosheets in different cases. **i** The fluorescence intensity of SOSG (in H_2_O) in presence of the DR-CoMo-LDH nanosheets under 1567 nm laser irradiation (0.5 W cm^−2^) and Rose Bengal under 550 nm laser irradiation (0.5 W cm^−2^). Source data are provided as a Source Data file.

The activities of as-prepared ultrathin 2D LDH nanosheets for generation of ROS under NIR laser irradiation were explored using singlet oxygen sensor green (SOSG) as a detector (fluorescence signal at 526 nm). The pristine CoMo-LDH nanosheets exhibit very poor activity toward the ROS generation under the irradiation of the 1567 nm laser, as evidenced by the SOSG indicator (Fig. 3a). Promisingly, its activity towards the ROS generation can be significantly enhanced by the simple acid treatment. For example, after etching the CoMo-LDH nanosheets at pH 4.0 for 2 h, the obtained DR-CoMo-LDH nanosheets exhibit excellent performance toward the ROS generation, which is about 97 times of the pristine CoMo-LDH nanosheets (insert in Fig. 3a). The activities of the DR-CoMo-LDH nanosheets towards the ROS generation under different-wavelength laser irradiation (808 nm, 1064 nm, 1270 nm, 1567 nm) were first evaluated. As shown in Supplementary Fig. 3, the results suggested that all the four types of lasers can excite the DR-CoMo-LDH nanosheets to generate ^1^O_2_ and its activity for ^1^O_2_ generation is highly dependent on the excitation wavelength. The generation of ^1^O_2_ increases with the increase of excitation wavelength and the DR-CoMo-LDH nanosheets exhibit the best performance under the 1567 nm laser irradiation. Such a long-wavelength NIR light can minimize tissue scattering with a penetration depth more than one centimeter^50^. The electron spin resonance (ESR) spectroscopy was also utilized to directly capture ^1^O_2_ by using 2,2,6,6-tetramethyl-4-piperidone (TEMP) as a spin probe. After being exposed to four types of laser irradiation, the characteristic ^1^O_2_ (1:1:1) signal could be clearly observed (Fig. 3b). Similarly, the DR-CoMo-LDH nanosheets present the best performance under the 1567 nm laser irradiation. Based on this result, we choose the 1567 nm laser as the excitation light source in the subsequent experiments. We also systematically explored the effects of different etching conditions, including pH values (7.0, 6.0, 5.0 and 4.0) and etching times (0.5, 1, 2, 4, 8, and 24 h), on the ROS generation performance of the DR-CoMo-LDH nanosheets. As shown in Supplementary Figs. 4 and 5, gradual increment in the fluorescence intensity of SOSG was observed for the DR-CoMo-LDH nanosheets after etching at pH 5.0 and 4.0 under 1567 nm laser irradiation. Interestingly, the DR-CoMo-LDH nanosheets etched for 2 h exhibit the strongest fluorescence intensity in two pH environments. Then we compared the ROS generation performance of DR-CoMo-LDH nanosheets etched at pH 7.0, 6.0, 5.0 and 4.0 for 2 h (Supplementary Fig. 6, and Fig. 3c). The results showed that the DR-CoMo-LDH nanosheets etched at pH 4.0 display the best ^1^O_2_ generation capacity. The generation of ^1^O_2_ in different cases was similarly monitored by using 9,10-anthracenediyl-bis(methylene) dimalonic acid (ABDA, an ^1^O_2_ indicator). As displayed in Supplementary Fig. 7 and Fig. 3d, no absorbance decreases of the ABDA solution containing DR-CoMo-LDH nanosheets etched at pH 7.0 are observed under 1567 nm laser irradiation. The weak acidic environment (pH 6.0 and 5.0) accelerated this process with a decreased absorbance of 9.3% and 20.5%, respectively. In the case of DR-CoMo-LDH nanosheets etched at pH 4.0, a decrease of 81.9% in absorbance is observed within 6 min, indicating the optimal photodynamic performance under this condition. This interesting feature of DR-CoMo-LDH nanosheets was further validated by TEMP probe in ESR spectroscopy (Fig. 3e).

### Catalytic activity of CoMo-LDH for O_2_ production

Subsequently, the catalytic activity of the DR-CoMo-LDH nanosheets to produce O_2_ in presence of H_2_O_2_ was systematically explored. As presented in Supplementary Fig. 8, as compared with the solution of DR-CoMo-LDH without H_2_O_2_, obvious gas bubbles can be observed from the solution of DR-CoMo-LDH with H_2_O_2_ at pH 7.4 and 6.5 incubated for 10 min. To confirm the gas bubbles were O_2_, a dissolved oxygen meter was applied to monitor the O_2_ concentration in H_2_O_2_ solution. It was found that O_2_ concentration in the DR-CoMo-LDH solution at pH 7.4 and 6.5 was quickly increased to 6.88 mg L^−1^ and 12.48 mg L^−1^, respectively after the addition of H_2_O_2_. In contrast, no significant O_2_ production was observed in the DR-CoMo-LDH solution without H_2_O_2_ (Fig. 3f). Such result clearly demonstrates the excellent catalytic activity of the DR-CoMo-LDH nanosheets towards the O_2_ generation by decomposing H_2_O_2_. To evaluate the effect of O_2_ on ROS generation performance, the ABDA indicator was utilized to detect ^1^O_2_ generation of DR-CoMo-LDH in different environments (hypoxia, normoxia, and normoxia + H_2_O_2_) under the 1567 nm laser irradiation. As show in Supplementary Fig. 9 and Fig. 3g, the DR-CoMo-LDH nanosheets in hypoxia environment did not attenuate the absorbance of ABDA, while the DR-CoMo-LDH nanosheets in normoxia environment attenuated 81.9% of ABDA, indicating large amounts of ^1^O_2_ generation in the presence of O_2_. After addition of H_2_O_2_, the DR-CoMo-LDH nanosheets in normoxia environment attenuated 92.7% of ABDA, demonstrating the highly efficient O_2_ generation ability of the DR-CoMo-LDH nanosheets in the presence of H_2_O_2_. The ^1^O_2_ measurement result in different cases using TEMP probe was consistent with that of ABDA (Fig. 3h). Therefore, the DR-CoMo-LDH nanosheets are expected to decompose H_2_O_2_ in the tumor microenvironment to provide sufficient oxygen source for ROS production under the NIR-III laser irradiation. To assess the ^1^O_2_ quantum yield, the Rose Bengal (RB) was adopted as a standard PS, and the relative quantum yield of the DR-CoMo-LDH nanosheets for ^1^O_2_ generation was calculated to be up to 87.5% (Fig. 3i and Supplementary Fig. 10).

### Characterization of structure changes

**Fig. 4:**
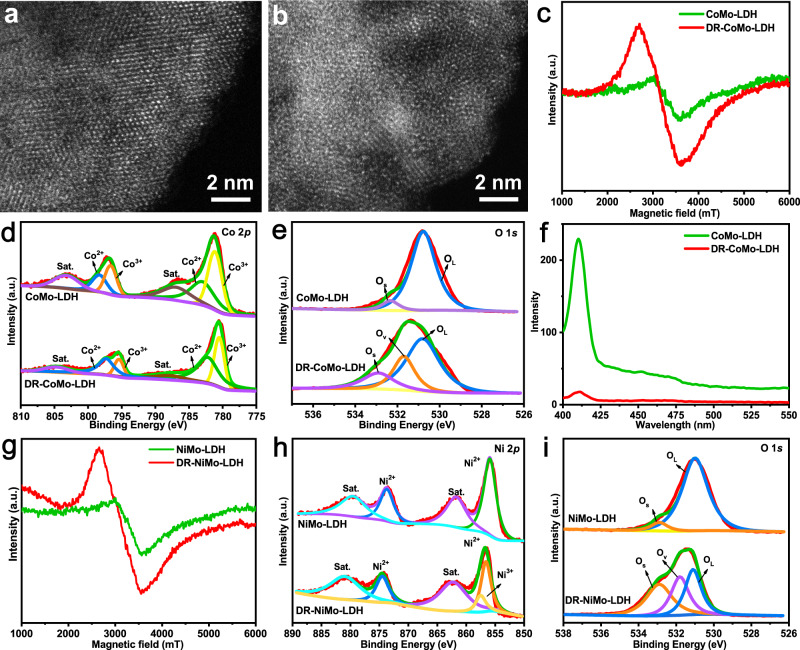
Fine-structure characterizations of CoMo-LDH and DR-CoMo-LDH nanosheets. Atomic-resolution STEM images of a typical **a** CoMo-LDH and **b** DR-CoMo-LDH nanosheet. Each experiment was repeated three times with similar results. **c** ESR spectra of the CoMo-LDH and DR-CoMo-LDH nanosheets. **d** Co 2*p* and **e** O 1*s* XPS spectra of CoMo-LDH and DR-CoMo-LDH nanosheets. **f** PL emission spectra of CoMo-LDH and DR-CoMo-LDH nanosheets. **g** ESR spectra of NiMo-LDH and DR-NiMo-LDH nanosheets. **h** Ni 2*p* and **i** O 1*s* XPS spectra of NiMo-LDH and DR-NiMo-LDH nanosheets. Source data are provided as a Source Data file.

To explore the structure changes induced by the acid treatment, the CoMo-LDH nanosheets before and after etching were characterized by XRD, TEM, atomic-resolution scanning transmission electron microscopy (STEM), ESR, X-ray photoelectron spectroscopy (XPS) and UV–vis–NIR diffuse reflection spectrum. In Supplementary Fig. 11a, the XRD pattern demonstrates that the crystallinity of the DR-CoMo-LDH nanosheets is significantly lower than that of the CoMo-LDH nanosheets (Fig. 2f). The TEM image shows that the DR-CoMo-LDH nanosheets maintain the ultrathin structure of CoMo-LDH nanosheets with a size of 50–80 nm (Supplementary Fig. 11b). In Fig. 4a, the atomic-resolution STEM image of the CoMo-LDH nanosheets presents single-crystalline structure with very few defects. In contract, the atomic-resolution STEM image shows that the DR-CoMo-LDH nanosheet is dominated by amorphous structures with small crystalline domains (Fig. 4b). Moreover, the ESR spectra also show that the DR-CoMo-LDH nanosheets give a strong signal at *G* = 2.2 than that of the CoMo-LDH nanosheets (Fig. 4c), indicating that rich defects are generated through the acid etching^51^. As shown in Supplementary Fig. 12a, peaks originating from Co, Mo and O were found both on the survey spectra of the CoMo-LDH and DR-CoMo-LDH nanosheets. In Fig. 4d, the both the XPS Co 2*p* spectra show four peaks at 783.21, 798.32, 781.20, and 796.66 eV, which can be attributed to Co^2+^ 2*p*_3/2_, Co^2+^ 2*p*_1/2_, Co^3+^ 2*p*_3/2_, Co^3+^ 2*p*_1/2_, respectively, proving the coexistence of Co^2+^ and Co^3+^ in the CoMo-LDH and DR-CoMo-LDH nanosheets. Interestingly, the Co^2+^/Co^3+^ ratio in CoMo-LDH nanosheets was 9:5, which changed to be 5:7 after etching. The XPS analysis of O 1 *s* is presented in Fig. 4e and the results indicate the existence of different oxygen species in CoMo-LDH and DR-CoMo-LDH nanosheets. Compared with the binding energies located at 530.78 eV (O_L_: lattice oxygen) and 532.91 eV (O_S_: adsorbed oxygen) in CoMo-LDH nanosheets, the peak at 531.71 eV indicates the presence of rich oxygen vacancies (OVs) in the DR-CoMo-LDH nanosheets. As observed in the XPS Mo 3*d* spectra (Supplementary Fig. 12b), both the CoMo-LDH and DR-CoMo-LDH nanosheets exhibit characteristic peaks located at 235.26 eV (3*d*_3/2_) and 232.14 eV (3*d*_5/2_), indicating that the etching has no significant effect on the Mo element. Therefore, the etching of CoMo-LDH nanosheets can effectively create OVs and cause changes in the valence states of Co element. In addition, it has been reported that Co^2+^ possesses the ability to catalyze the generation of ·OH through H_2_O_2_, that is, it can achieve chemodynamic therapy (CDT)^52^. In this regard, we compared the CDT and PDT performance of the DR-CoMo-LDH nanosheets with 2′,7′-dichlorofluorescein diacetate (DCFH-DA) as a ROS probe. It can be seen from Supplementary Fig. 13 that the DR-CoMo-LDH nanosheets produced very small amount of ROS in the presence of H_2_O_2_, while the ROS produced under 1567 nm laser irradiation increased significantly, indicating that the CDT performance of the DR-CoMo-LDH nanosheets is negligible compared with its PDT performance.

The band structure of the CoMo-LDH and DR-CoMo-LDH nanosheets were determined by UV–vis–NIR diffuse reflectance spectra. As presented in Supplementary Fig. 14, the bandgaps (*E*_g_) of the CoMo-LDH and DR-CoMo-LDH nanosheets are calculated to be 1.70 eV and 0.54 eV, respectively, through the intercept of tangent with abscissa. The narrow *E*_g_ allows the DR-CoMo-LDH to be excited by low-energy light in the NIR-III window. Subsequently, the conduction band (CB) values of CoMo-LDH and DR-CoMo-LDH nanosheets are determined as −0.86 and −1.02 eV, respectively, by extrapolating the Mott–Schottky plot to 1/*C*^2^ = 0 (Supplementary Fig. 15a). Then, the valence band (VB) values were calculated to be 0.84 and −0.48 eV, respectively. The energy band and electrons transfer are schematically illustrated in Supplementary Fig. 15b. Under laser irradiation, the electron-hole pairs in the VB of the DR-CoMo-LDH nanosheets with a low *E*_*g*_ of 0.54 eV were easily excited and separated, with electrons and holes occupying the CB and VB, respectively. The photoelectrons are ejected to surroundings and react with O_2_ to produce intermediate ·O_2_^−^, which can further combine with holes to form the final ^1^O_2_^53–55^. ESR spectroscopy demonstrated the generation of intermediate ·O_2_^−^ for DR-CoMo-LDH sample (Supplementary Fig. 16). The generation of ·O_2_^−^ was further confirmed using superoxide kit assay (dihydrorhodamine 123: DHR123)^56^, in which the probe fluorescence peak at 526 nm can be turned on after reacting with ·O_2_^−^. Similar to the ESR analysis, a strong fluorescence intensity of DHR123 was observed in presence of DR-CoMo-LDH under 1567 nm irradiation, while nearly no fluorescence was detected in the presence of CoMo-LDH (Supplementary Fig. 17).

The catalytic activities of CoMo-LDH and DR-CoMo-LDH nanosheets to generate ·OH under 1567 nm irradiation were also explored using terephthalic acid (TA) as a ·OH specific probe, which can combine with ·OH to produce fluorescent 2-hydroxy-terephalic acid (TAOH) with a characteristic fluorescence emission at 435 nm. As indicated in Supplementary Fig. 18a,b, no fluorescence signal was found for the TA probe in presence of CoMo-LDH and DR-CoMo-LDH nanosheets under 1567 nm irradiation, indicating that both of them cannot catalyze to generate ·OH under 1567 nm irradiation. Methylene blue (MB) was also chosen as a probe, which can be degraded by ·OH. Similarly, there was no obvious degradation of MB under the treatment of CoMo-LDH and DR-CoMo-LDH nanosheets with 1567 nm irradiation (Supplementary Fig. 18c, d), further demonstrating no ·OH generation.

It has been widely reported that introducing OVs as the intrinsic defects in metal oxides or metal hydroxides can effectively improve the charge separation efficiency, because OVs as electron donors can increase the majority carrier density^57–59^. Meanwhile, OVs can provide photoinduced charge traps to reduce the electron-hole recombination^60,61^. In view of this, the photoluminescence (PL) properties of CoMo-LDH and DR-CoMo-LDH nanosheets were investigated by PL emission spectra, since the PL behavior derived from the recombination of photogenerated electron-hole pairs can reflect the separation of photoinduced charge carriers. As shown in Fig. 4f, the CoMo-LDH nanosheets displayed strong PL emission peaks at 413 nm, which is attributed to the charge transfer transition. The PL intensity of this peak decreased significantly in the DR-CoMo-LDH nanosheets, indicating a significantly suppressed radiative recombination of photogenerated electron-hole. The suppression of the radiative recombination should be beneficial to promote ROS generation since the photoelectrons are effectively utilized to produce ·O_2_^−^, which further combines with holes to form the final ^1^O_2_.

### Defect engineering of NiMo-LDH and its catalytic activity

Interestingly, our simple defect engineering induced by acid treatment can be also used to etch ultrathin 2D NiMo-LDH nanosheets to obtain defect-rich NiMo-LDH nanosheets (denoted as DR-NiMo-LDH). The crystal structure of DR-NiMo-LDH nanosheets was investigated by XRD (Supplementary Fig. 19a), and the results demonstrated the much-decreased crystallinity compared to NiMo-LDH nanosheets (Fig. 2i). As shown in the TEM image, the DR-NiMo-LDH nanosheets have an ultrathin thickness similar to NiMo-LDH nanosheets with a size of 40–80 nm (Supplementary Fig. 19b). In Fig. 4g, similar to the DR-CoMo-LDH nanosheets, obvious defect signal in DR-NiMo-LDH was detected from ESR spectroscopy. The element valence states of the NiMo-LDH nanosheets before and after etching were investigated by XPS. As illustrated in Supplementary Fig. 20a, the XPS analysis revealed Ni 2*p*, O 1*s*, and Mo 3*d* peaks. According to the high-resolution XPS scans of Mo 3*d* (Supplementary Fig. 20b), the peak positions at 235.46 eV (Mo^6+^ 3*d*_3/2_) and 232.34 eV (Mo^6+^ 3*d*_5/2_) were basically identical before and after etching; however, the peak intensity of Mo^6+^ in DR-NiMo-LDH decreased significantly, manifesting that partial Mo^6+^ ions were removed by etching. In the Ni 2*p* spectrum (Fig. 4h), there existed two main peaks at 856.04 eV (Ni 2*p*_3/2_) and 873.54 eV (Ni 2*p*_1/2_) as well as two satellite peaks (861.54 eV and 880.84 eV), which demonstrated the high-spin Ni^2+^ state in NiMo-LDH. After etching, a new peak of 857.51 eV appeared in the DR-NiMo-LDH nanosheets, proving the formation of Ni^3+^ state. In Fig. 4i, the binding energy of OVs only presented in the DR-NiMo-LDH nanosheets, indicating the efficient generation of OVs after etching. The absorption spectrum of the DR-NiMo-LDH nanosheets was determined by an UV–vis–NIR diffuse spectrophotometer and it gives strong absorption in the NIR region (1000–1600 nm) (Supplementary Fig. 21). Subsequently, the photodynamic properties of NiMo-LDH before and after etching were evaluated using SOSG as a detector under 1567 nm laser irradiation (0.5 W cm^−2^). As displayed in Supplementary Fig. 22a–c, the fluorescence intensity of SOSG in DR-NiMo-LDH nanosheets is much stronger than that of NiMo-LDH nanosheets, revealing that defect engineering of NiMo-LDH nanosheets induced by acid etching could significantly increase the production yield of ^1^O_2_. This result was further confirmed by ESR spectroscopy using TEMP as a spin probe. After being exposed to 1567 nm laser irradiation for 6 min, the characteristic ^1^O_2_ (1:1:1) signal could be clearly observed in DR-NiMo-LDH rather than NiMo-LDH (Supplementary Fig. 23), which was consistent with the results tested using the SOSG as the probe.

### Surface modification

The aforementioned results have proven that the DR-CoMo-LDH nanosheets can be used as a highly active PS for PDT in the NIR-III window. Therefore, the DR-CoMo-LDH nanosheets were modified with PEG (denoted as DR-CoMo-LDH-PEG) to improve its biocompatibility. The Fourier transform infrared (FT-IR) spectra was performed to confirm the PEGylation of DR-CoMo-LDH nanosheets. As shown in Supplementary Fig. 24, the characteristic absorption peak at 950 cm^−1^ (C–O–C stretching vibration) of PEG is observed in the DR-CoMo-LDH-PEG, accompanied with the characteristic bands of LDH at 3433 cm^−1^ (O−H stretching vibration) and 1354 cm^−1^ (vibration absorption peak of C = O in CO_3_^2−^), proving the successful surface modification with PEG. The dynamic light scattering (DLS) technique displayed the unchanged hydrodynamic size of the DR-CoMo-LDH (86 ± 4 nm) and DR-CoMo-LDH-PEG (91 ± 5 nm) within two weeks (Supplementary Fig. 25), indicating the excellent dispersion stability. From the thermogravimetric (TG) analysis, the weight percentage of PEG in DR-CoMo-LDH-PEG is measured to be ≈23.2% (Supplementary Fig. 26). Additionally, the zeta potentials of the CoMo-LDH, DR-CoMo-LDH and DR-CoMo-LDH-PEG nanosheets were measured. As revealed in Supplementary Fig. 27, the zeta potential of the CoMo-LDH nanosheets is 12.6 ± 2.4 mV, and it becomes −16.7 ± 2.2 mV after acid etching. The zeta potentials of the DR-CoMo-LDH-PEG nanosheets in water, high glucose Dulbecco’s modified Eagles medium (DMEM) and phosphate-buffered saline (PBS) are close to that of the DR-CoMo-LDH nanosheets with electronic potential of −16.4 ± 2.5, −17.3 ± 2.6 and −16.3 ± 2.7 mV, respectively. The isothermal titration calorimetry (ITC) measurements were carried out to investigate the interaction between DR-CoMo-LDH and PEG. The thermodynamic parameters show that the entropy change (∆*S*) and enthalpy change (∆*H*) are both negative in DR-CoMo-LDH-PEG system (Supplementary Fig. 28), indicating the presence of hydrogen bonding or Van der Waals’ force between DR-CoMo-LDH and PEG^37,62^.

### In vitro study with 4T1 cells

Based on the above exciting results, the in vitro cell tests were further performed and the biocompatibility of DR-CoMo-LDH-PEG was accessed in 4T1, MREpiC, and Cos-7 cells by 3-(4,5-dimethylthiazol-2-yl)-2,5-diphenyltetrazolium bromide (MTT) assay. Cell viability results demonstrate that DR-CoMo-LDH-PEG shows negligible cytotoxicity to 4T1, Cos-7 and MREpiC cells even at concentrations up to 200 µg mL^−1^ (Supplementary Fig. 29). To further verify the biocompatibility of the DR-CoMo-LDH-PEG nanosheets, the hemolysis assay was performed on mouse red blood cells (RBCs) (Supplementary Fig. 30). H_2_O and PBS were chosen as positive and negative controls, respectively, since RBCs will be lysed under hyperosmosis in H_2_O but not in PBS. After incubation with RBCs for 4 h, the hemolysis rates of the DR-CoMo-LDH-PEG nanosheets with various concentrations (12.5, 25, 50, 100, 200 μg mL^−1^) are lower than the international standard (5%), indicating no obvious hemolytic activity of the DR-CoMo-LDH-PEG nanosheets.

**Fig. 5:**
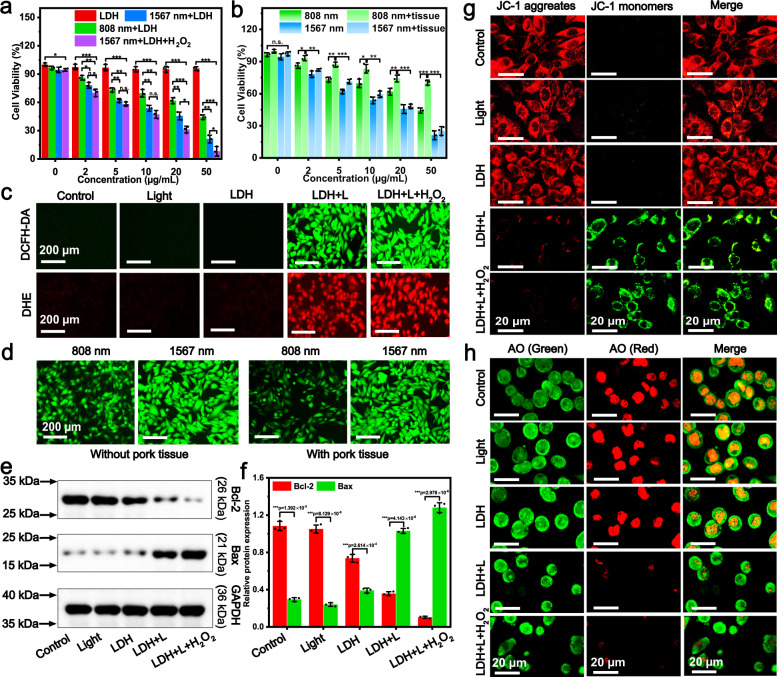
In vitro PDT effects of DR-CoMo-LDH-PEG with 4T1 cells. **a** and **b** In vitro cytotoxicity profiles of 4T1 cells incubated with DR-CoMo-LDH-PEG under different conditions. Data are presented as mean values ± s.d. (*n* = 3). Statistical analysis was performed via one-way ANOVA. *p < 0.05, **p < 0.01, ***p < 0.001. **c** ROS fluorescence probe DCFH-DA and ·O_2_^−^ fluorescence probe DHE staining images of 4T1 cells incubated with DR-CoMo-LDH-PEG in different cases: 1) control, 2) 1567 nm laser (0.5 W cm^−2^ for 6 min), 3) DR-CoMo-LDH-PEG, 4) DR-CoMo-LDH-PEG + 1567 nm laser, 5) DR-CoMo-LDH-PEG + 1567 nm laser + H_2_O_2_ (100 μM). Each experiment was repeated three times with similar results. **d** DCFH-DA staining images of cells after incubation with DR-CoMo-LDH-PEG under 808 nm and 1567 nm laser without/with pork tissue. Each experiment was repeated three times with similar results. **e** Western blotting analysis of Bcl-2 and Bax protein expression level of 4T1 cells 8 h after treatment of different groups. GAPDH was used as loading control. **f** Light intensities of Bcl-2 and Bax proteins expression as the ratio of the corresponding protein to GAPDH from WB results. Data are presented as mean values ± s.d. (*n* = 3). Statistical analysis was performed via one-way ANOVA. ***p < 0.001. **g** Mitochondrial membrane depolarization of cells and **h** CLSM images of AO staining for lysosome after various treatments: control, light, DR-CoMo-LDH-PEG, DR-CoMo-LDH-PEG + light, DR-CoMo-LDH-PEG + light + H_2_O_2_. Each experiment was repeated three times with similar results. Source data are provided as a Source Data file.

The cellular uptake of DR-CoMo-LDH-PEG was also studied on 4T1 cells, and the intense red fluorescence signal from Rhodamine B (RhB)-labeled DR-CoMo-LDH-PEG was found in the cells at 24 h (Supplementary Fig. 31), verifying the effective intracellular internalization of DR-CoMo-LDH-PEG via endocytosis. We next investigated in vitro PDT efficiency of the DR-CoMo-LDH-PEG under different conditions. As shown in the Fig. 5a, individual NIR irradiation (light) and light + H_2_O_2_ show unobvious cytotoxicity to 4T1 cells. However, when the cell was incubated with the DR-CoMo-LDH-PEG for 24 h and exposed to 6 min NIR irradiation, the cell viability gradually decreased as the concentration increase of the DR-CoMo-LDH-PEG. It is worth pointing out that compared with 808 nm irradiation, the 1567 nm irradiation can give rise to more cell deaths under the same experimental conditions, indicating that the 1567 nm irradiation can produce more ^1^O_2_ levels. Moreover, DR-CoMo-LDH-PEG + light + H_2_O_2_ group resulted in the best PDT efficiency due to O_2_ supply generated by the reaction of DR-CoMo-LDH-PEG and overexpressed intracellular H_2_O_2_. The intracellular O_2_ generation ability of the DR-CoMo-LDH-PEG nanosheets was studied by monitoring the hypoxia level of 4T1 cells after various treatments under hypoxic environment. The red hypoxia staining reagent was adopted as a hypoxia probe since it can emit red fluorescence after being degraded by nitro-reductase in hypoxic cells^63^. As presented in Supplementary Fig. 32, the intense fluorescence signal was observed both in blank group and DR-CoMo-LDH-PEG group. In contrast, the fluorescence signal intensity in DR-CoMo-LDH-PEG + H_2_O_2_ group significantly decreased, suggesting the hypoxia relief by the DR-CoMo-LDH-PEG nanosheets in the presence of H_2_O_2_ due to O_2_ generation.

In order to further demonstrate the advantage of NIR-III irradiation, we investigated the PDT performance under 808 and 1567 nm irradiation when the light source was blocked by 5 mm pork slice. As shown in Fig. 5b, no obvious difference of cell viability for DR-CoMo-LDH-PEG + 1567 nm irradiation was found with or without a pork slice, however, the PDT efficiency of DR-CoMo-LDH-PEG with the pork slice decreased obviously under 808 nm irradiation with a pork slice, which demonstrates the excellent tissue penetrability of the 1567 nm laser.

The efficiency of the DR-CoMo-LDH nanosheets for in vitro PDT was further confirmed by live/dead staining experiments with calcein acetoxymethyl ester (Calcein-AM) and propidium iodide (PI). No red fluorescence was observed for control, light and DR-CoMo-LDH-PEG groups (Supplementary Fig. 33). In contrast, DR-CoMo-LDH-PEG + light and DR-CoMo-LDH-PEG + light + H_2_O_2_ groups presented high percentage of dead 4T1 cells, which was consistent with the MTT assay results. Flow cytometry analysis further demonstrated DR-CoMo-LDH-PEG + light and DR-CoMo-LDH-PEG + light + H_2_O_2_ groups induced obvious PDT-related cell apoptosis (Supplementary Fig. 34). Moreover, intracellular ROS level was measured using DCFH-DA as a ROS detector. There was no 2’,7’-dichlorofluorescein (DCF) fluorescence detected in 4T1 cells incubated with PBS, DR-CoMo-LDH-PEG or individual 1567 nm irradiation. However, with 1567 nm laser irradiation, DR-CoMo-LDH-PEG induce obvious green florescence enhancement, while DR-CoMo-LDH-PEG + H_2_O_2_ could further increase this fluorescence intensity. Meanwhile, remarkable ·O_2_^−^ generation can be found in the DR-CoMo-LDH-PEG + light and DR-CoMo-LDH-PEG + light + H_2_O_2_ groups, which was similar to the DCFH-DA results (Fig. 5c). In addition, DR-CoMo-LDH-PEG + 1567 nm irradiation showed slightly green florescence difference with or without pork slice. However, the DCF fluorescence intensity of DR-CoMo-LDH-PEG + 808 nm irradiation decreased dramatically with pork slice (Fig. 5d). The intracellular ROS and ·O_2_^−^ levels determined by flow cytometry analysis (Supplementary Fig. 35) show the similar results in Fig. 5c, d. In previously reported work, thicker tissue was used to mimic the penetration of light more accurately^64,65^. In view of this, pork slices with different thicknesses (0, 2, 5, 10 mm) were further utilized as barriers to evaluate the penetration ability of 1567 nm laser. As shown in Supplementary Fig. 36, under the cover of pork slices with different thickness, there was no significant difference in the cytotoxicity and ROS levels of DR-CoMo-LDH-PEG under 1567 nm irradiation, demonstrating the remarkable penetration capability of 1567 nm laser, which is conducive to the treatment of deep tumors.

To compare the in vitro photoactivities of DR-CoMo-LDH-PEG and DR-NiMo-LDH-PEG nanosheets, MTT assay was first conducted to evaluate their cytotoxicity toward 4T1 cells under 1567 nm irradiation. The results in Supplementary Fig. 37a show that the cytotoxicity of DR-CoMo-LDH-PEG nanosheets was significantly higher than that of DR-NiMo-LDH-PEG nanosheets, indicating the better photoactivities of DR-CoMo-LDH-PEG nanosheets. Afterward, DCFH-DA probe was employed to visualize ROS levels. In Supplementary Fig. 37b, 4T1 cells incubated with DR-CoMo-LDH-PEG nanosheets exhibited the most intensive fluorescent signals of DCF after 1567 nm irradiation, in comparison with the control, light, and DR-NiMo-LDH-PEG nanosheets groups, which again verified the much stronger photoactivities of DR-CoMo-LDH-PEG nanosheets.

Based on the above preliminary results, western blotting (WB) analysis was carried out to evaluate the expression of antiapoptotic (Bcl-2) and apoptotic (Bax) proteins with glyceraldehyde-3-phosphate dehy-drogenase (GAPDH) as a control. As presented in Fig. 5e, f, compared with 4T1 cells treated with DR-CoMo-LDH-PEG or light, a significantly increased Bax protein and decreased Bcl-2 protein is observed from cells treated with DR-CoMo-LDH-PEG + light and DR-CoMo-LDH-PEG + H_2_O_2_ + light. In particular, a higher Bax protein level and lower Bcl-2 protein level is induced by DR-CoMo-LDH-PEG + H_2_O_2_ + light group than DR-CoMo-LDH-PEG + light group, since the O_2_ generated by H_2_O_2_ decomposition improves the generation of ROS, further promoting cell apoptosis. The above results were consistent with that of MTT assay. Then, mitochondrial dysfunction was assessed by JC-1 fluorescent probe, which can reflect the mitochondrial membrane potential and ROS-mediated cell apoptosis behavior. No obvious green fluorescence was observed for control, light and DR-CoMo-LDH-PEG group alone, while DR-CoMo-LDH-PEG + light led to weak red fluorescence and strong green fluorescence, suggesting mitochondrial depolarization induced by PDT. Furthermore, no red fluorescence and bright green fluorescence intensity was found in DR-CoMo-LDH-PEG + light + H_2_O_2_ group, indicating more profound mitochondrial permeability transition and severer oxidative stress injury with increasing ^1^O_2_ levels (Fig. 5g). We further utilized acridine orange (AO, the lysosomal integrity indicator) to evaluate the PDT-mediated lysosome destruction. For control, light, and DR-CoMo-LDH-PEG groups, a large amount of red fluorescence of AO was found, indicating the integrity of lysosomal compartments. However, for DR-CoMo-LDH-PEG + light and DR-CoMo-LDH-PEG + light + H_2_O_2_ groups, the disappearance of AO red fluorescence suggested severe disruption of the integrity of lysosomes (Fig. 5h), demonstrating severe oxidative damage to 4T1 cells.

### In vivo cancer therapy

**Fig. 6:**
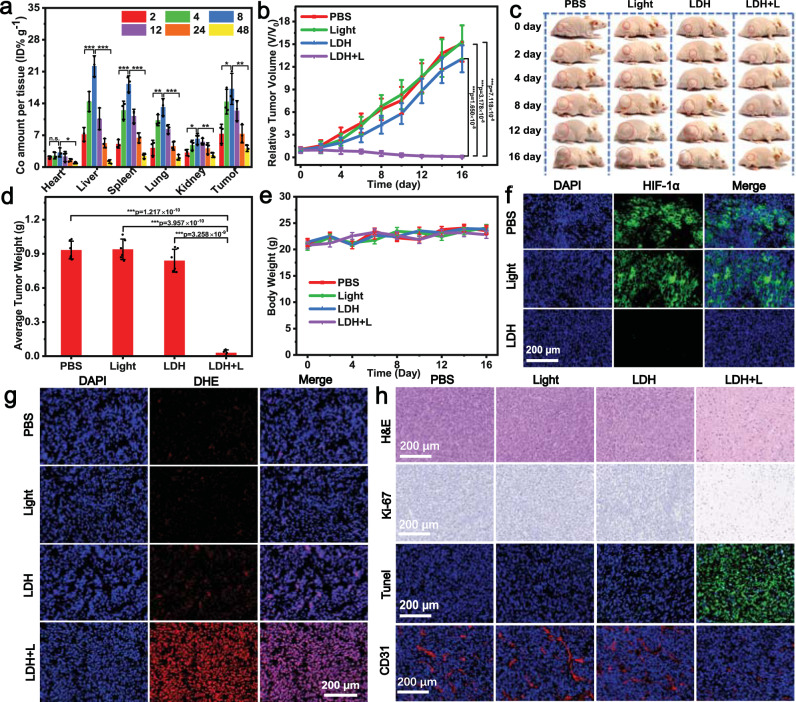
In vivo PDT effects of DR-CoMo-LDH-PEG conducted on 4T1 tumor-bearing mice. **a** Biodistribution of DR-CoMo-LDH-PEG in mice by monitoring the Co concentration at various time points post-injection. Data are presented as mean values ± s.d. (*n* = 3). Statistical analysis was performed via one-way ANOVA. *p < 0.05, **p < 0.01, ***p < 0.001. **b** Tumor growth curves of mice after various treatments. Data are presented as mean values ± s.d. (*n* = 6). Statistical analysis was performed via one-way ANOVA. ***p < 0.001. **c** Representative photographs of mice with treatments at various time points and **d** corresponding average weight of tumors taken on Day 16. Data are presented as mean values ± s.d. (*n* = 6). Statistical analysis was performed via one-way ANOVA. ***p < 0.001. **e** Body weight change of 4T1 tumor-bearing mice after different treatments. Data are presented as mean values ± s.d. (*n* = 6). **f** HIF-1α, **g** DHE, and **h** H&E, Ki-67, TUNEL and CD31 staining assay of tumor slices from various groups of mice after 16 days of treatment: 1) PBS, 2) 1567 nm laser (0.5 W cm^−2^ for 6 min), 3) DR-CoMo-LDH-PEG, 4) DR-CoMo-LDH-PEG + 1567 nm laser. Each experiment was repeated three times with similar results. Source data are provided as a Source Data file.

Inspired by the exciting in vitro PDT results, in vivo PDT anti-cancer activity of DR-CoMo-LDH-PEG was then conducted on 4T1 tumor-bearing mice. First, the blood circulation half-life of DR-CoMo-LDH-PEG was calculated to be 0.57 (t_1/2(α)_) and 10.05 h (t_1/2(β)_) by secondary exponential fitting (Supplementary Fig. 38). The long blood circulation time was beneficial for the accumulation of DR-CoMo-LDH-PEG at the tumor site. The biodistribution of the DR-CoMo-LDH-PEG in the major organs (heart, liver, spleen, lung, and kidney) and tumor tissues were also investigated (Fig. 6a). The major organs and tumors were collected at indicated time points (1–48 h) for inductively coupled plasma-atomic emission spectroscopy (ICP-AES) analysis. The results revealed that DR-CoMo-LDH-PEG tended to be accumulated at liver and spleen, and could also significantly enrich into tumor tissues, where the maximum accumulation occurred at 8 h post injection.

4T1 tumor-bearing mice were divided into four groups randomly when the tumor size reached 80 mm:^3^ PBS, NIR-III light irradiation (light), DR-CoMo-LDH-PEG, and DR-CoMo-LDH-PEG + light. For mice treated with laser irradiation, they were exposed to the 1567 nm light with the power density of 0.5 W cm^−2^ for 6 min at 8 h post intravenous injection. Considering that NIR-III light irradiation can induce a photothermal effect due to the absorption of 1567 nm light at the tumor tissue, the tumors were wiped with alcohol cotton for 15 s after every 1 min of light irradiation to avoid the potentially temperature rise. As shown in Supplementary Fig. 39, the temperature of the tumors of mice in the light and DR-CoMo-LDH-PEG + light group increased no more than 5 °C, indicating that no photothermal effect occurred. The tumor growth kinetics showed that the control and NIR-III light irradiation group exhibited the obvious tumor growth (Fig. 6b). The tumor growth of mice injected with DR-CoMo-LDH-PEG could be slightly inhibited due to the weak CDT performance. Specifically, the group treated with DR-CoMo-LDH-PEG + light showed complete tumor regression. The representative photos of corresponding mice were displayed in Fig. 6c. After 16 days of treatment, all tumor tissues were collected and weighted. The digital photographs (Supplementary Fig. 40) and the average weight (Fig. 6d) of tumors further verified that DR-CoMo-LDH-PEG + light can efficaciously inhibit the tumor growth. No obvious body weight changes for all groups were observed during treatment (Fig. 6e).

We further evaluated the in vivo O_2_ generation ability of DR-CoMo-LDH-PEG. Tumor hypoxia was then examined with hypoxia-inducible factor (HIF)-1α staining (green signals). Results suggested DR-CoMo-LDH-PEG can significantly relieve hypoxia (Fig. 6f). To determine the generation of ROS in tumor tissues, dihydroethidium (DHE) staining probe was adopted to stain tumor slices. As presented in Fig. 6g, prominent red fluorescence was found in tumors treated with DR-CoMo-LDH-PEG + light, confirming large amounts of ROS generation. Subsequently, hematoxylin and eosin (H&E) staining assays of tumor slices were performed to verify the therapeutic efficiency of DR-CoMo-LDH-PEG (Fig. 6h). Among four groups, mice administrated with DR-CoMo-LDH-PEG + light suffered the most significant tumor tissue damage, demonstrating its excellent PDT performance. Additionally, Ki-67 and terminal deoxynucleotidyl transferase (TdT)-mediated deoxyuridine triphosphate (dUTP) nick end labeling (TUNEL) staining assays were performed to further evaluate the cancer cells proliferation and apoptosis levels, respectively. The DR-CoMo-LDH-PEG + light group had the fewest brown area, validating the excellent inhibiting effect of DR-CoMo-LDH-PEG on the proliferation of cancer cells. Similarly, the positive TUNEL staining signal was highest in the DR-CoMo-LDH-PEG + light group, which was coincident with the results of H&E and Ki-67 staining. Meanwhile, the formation of platelet endothelial cell adhesion molecule-1 (CD31)-positive vessels was also significantly suppressed after the treatment of DR-CoMo-LDH-PEG plus NIR irradiation (Fig. 6h).

The histological examination of major organs from mice injected with DR-CoMo-LDH-PEG and PBS showed little pathological abnormalities (Supplementary Fig. 41). Blood biochemical analysis was also conducted to assess long-term biosafety of DR-CoMo-LDH-PEG. The blood was collected at Day 1 and Day 16 after the administration of DR-CoMo-LDH-PEG. Kidney and liver function markers as well as blood cell counts were measured. The levels of all indexes at Day 1 and 16 showed no obvious change for the control and DR-CoMo-LDH-PEG groups, indicating negligible blood toxicity of DR-CoMo-LDH-PEG (Supplementary Fig. 42). Furthermore, to investigate the degradation and metabolism of the DR-CoMo-LDH-PEG nanosheets in vivo, Co concentrations in both urine and feces were measured by ICP-AES. As shown in Supplementary Fig. 43, a high level of Co was observed in the first 8 h and then declined gradually to a low level at 48 h, confirming that the DR-CoMo-LDH-PEG nanosheets could be metabolized and excreted by the way of urine and feces. Overall, the aforementioned results proved the excellent in vivo biocompatibility of DR-CoMo-LDH-PEG, potentiating its further application as a NIR-III PDT nanoagent.

## Discussion

The facile acid-etching strategy could alter the electronic structure of 2D LDH nanosheets, including the following two aspects: (1) narrowing the bandgap and (2) introducing a lot of discrete defect energy levels by creating a lot of OVs. The highly efficient PDT performance of DR-CoMo-LDH nanosheets could be attributed to electronic structure changing induced by the defect engineering. The electronic structure changing can boost the catalytic activity of 2D LDH nanosheets towards the ROS generation through the following points: (i) the narrow *E*_g_ allows the defect-rich LDH nanosheets to absorb longer wavelength light energy, and thus it can be excited by NIR-III 1567 nm laser irradiation; (ii) the increased carrier density induced by OVs and narrow *E*_g_ make the electrons in the VB easier to be excited to the CB, thus effectively improving the charge separation efficiency of the photogenerated electron-hole pairs; (iii) the rich discrete defect energy levels within the bandgap provided by OVs can capture electrons or holes and thus significantly suppress the radiative recombination of photogenerated electron-hole pairs. The suppression of the radiative recombination could significantly enhance the possibility of photogenerated electron-hole pairs to react with O_2_ for ROS generation. Therefore, the DR-CoMo-LDH nanosheets exhibit significantly enhanced activity (i.e., ~97 times) towards the ROS generation, thus making it an efficient PS for eliminating cancer cells in vitro and ablating tumors in vivo under the NIR-III 1567 nm laser irradiation.

In summary, we have achieved the defect engineering of hydrothermal-synthesized ultrathin 2D CoMo-LDH and NiMo-LDH nanosheets through a simple acid treatment, which can be used as highly active PSs for photodynamic cancer therapy in the NIR-III window. Importantly, this effective defect engineering strategy can be further extended to engineer 2D NiMo-LDH nanosheets and significantly enhance its subsequent activity towards ROS generation under the NIR-III laser irradiation. Our study has demonstrated that defect engineering is a simple but powerful strategy to activate LDH nanosheets for highly efficient NIR PDT. We believed that this defect engineering could be also an effective and promising strategy to improve the performance of ultrathin 2D LDHs in other biological applications, such as photothermal therapy, CDT and sonodynamic therapy. Moreover, defect engineering might be also used to tune other 2D materials, such as transition metal dichalcogenides, metal oxides and MXenes, for highly efficient NIR PDT.

## Methods

### Chemicals

Ammonium molybdate tetrahydrate ((NH_4_)_6_Mo_7_O_24_·4H_2_O, >99.0%), nickel nitrate hexahydrate (Ni(NO_3_)_2_·6H_2_O, >99.0%), cobalt nitrate hexahydrate (Co(NO_3_)_2_·6H_2_O, >99.0%), sodium hydroxide (NaOH, >98.0%), SOSG, TEMP, ABDA were obtained from Aladdin Reagent (Shanghai, China). Calcein-AM/PI, DCFH-DA and MTT were acquired from Sigma-Aldrich and Fisher (USA). PBS and TUNEL apoptosis detection kit were acquired from Beijing Solarbio Science & Technology Co., Ltd (China). DMEM, trypsin-EDTA (0.25%), fetal bovine serum (FBS), streptomycin and penicillin were obtained from Invitrogen Gibco (Carlsbad, CA). DHE, AO, and JC-1 were purchased from Shanghai Beyotime Biotechnology Co., Ltd (China). All chemicals used in this study were not further purified. The distilled (DI) water used in all processes came from the Millipore system.

### Characterizations

XRD patterns were performed using a Shimadzu XRD-6000. TEM images were captured via a JEM-2010 transmission electron microscope (200 kV accelerating voltage). The thickness of the CoMo-LDH nanosheets was characterized by AFM (MultiMode 8, Bruker). ESR spectra were acquired on the Bruker EMX1598 spectrometer. A JEOL ARM200F (JEOL, Tokyo, Japan) was used to record the STEM images. The element valence states of the CoMo-LDH nanosheets was investigated by XPS (Escalab 250Xi, Thermo Scientific, USA). Hydrodynamic sizes and zeta potentials were investigated by a Zetasizer UV spectrometer (Malvern Instruments, UK). The contents of Co were detected by ICP-AES (Shimadzu ICPS-7500). Cellular fluorescence images were performed on a confocal microscopy (Leica DM6000M, Germany). Fluorescence quantity analysis was conducted by Beckman Flow cytometer (USA).

### Preparation of CoMo-LDH and NiMo-LDH nanosheets

Firstly, 200 mL deionized (DI) water was continuously bubbled with N_2_ for at least 30 min under boiling condition. Then, (NH_4_)_6_Mo_7_O_24_·4H_2_O (5 mmol) and Co(NO_3_)_2_·6H_2_O (1 mmol) were dissolved in 70 mL of cooled DI water and stirred to obtain solution A. NaOH (2 g) was dissolved in another 50 mL DI water as the solution B. Next, the solution B was added dropwise to the solution A at 25 °C under N_2_ atmosphere until the pH of the solution was about 9.70. Thirdly, the resultant precipitation was transferred into a hydrothermal Teflon container (100 mL) and treated at 60 °C for 24 h. The resulting CoMo-LDH colloid was washed with DI water via centrifuge for four times. The NiMo-LDH colloid was synthesized via a similar procedure except changing the Co(NO_3_)_2_·6H_2_O (1 mmol) to Ni(NO_3_)_2_·6H_2_O (3 mmol) and reaction temperature to 80 °C.

### Preparation of DR-CoMo-LDH and DR-NiMo-LDH nanosheets

The as-synthesized CoMo-LDH (NiMo-LDH) colloid was dispersed in buffer solution at pH 4.0 for 2 h. The resultant DR-CoMo-LDH (DR-NiMo-LDH) was then collected by centrifugation (6000 × *g*, 5 min) and re-dispersed in cooled DI water.

### Preparation of DR-CoMo-LDH-PEG nanosheets

The PEG (0.06 mol) was added into the DR-CoMo-LDH solution with ultrasonication for 30 min and then stirred at 600 × *g* for 8 h. The obtained DR-CoMo-LDH-PEG was centrifuged for 5 min (6000 × *g*) and washed thoroughly with DI water three times.

### Detection of ROS

The ESR spectrometer was adopted to determine the ROS generation of CoMo-LDH in different cases by using TEMP as the ^1^O_2_ trapping agent. Briefly, 100 µL of TEMP (90 mM) was added to the CoMo-LDH solution (100 µL, 90 μg mL^−1^), and then ^1^O_2_ was detected by ESR under different laser irradiation for 6 min (808 nm, 1064 nm, 1270 nm and 1567 nm; 0.5 W cm^−2^). The ^1^O_2_ generation of CoMo-LDH etched at different pH (7.0, 6.0, 5.0, and 4.0) was detected by ESR under 1567 nm laser irradiation for 6 min (0.5 W cm^−2^). The parameters for ESR measurements are described as follows: the scan time is 41.9 s, the scan width is 200 G, the microwave power is 2.015 mW, and the microwave frequency is 9.873 GHZ.

The generation of ^1^O_2_ was further confirmed with SOSG and ABDA. DR-CoMo-LDH (200 μL, 300 μg mL^−1^) and SOSG (400 μL, 30 mM) were mixed together and then exposed to four types of laser irradiation for 6 min (0.5 W cm^−2^). The generated ^1^O_2_ was determined by monitoring the fluorescence signal at 526 nm. Each sample was monitored every 1 min with the slit width of 5.0 nm for excitation and emission. Similarly, after adding DR-CoMo-LDH (80 μL, 2.5 mg mL^−1^) and ABDA (50 μL, 7.5 mM) into 1870 μL H_2_O, the obtained solution was exposed to four types of laser irradiation for 6 min (0.5 W cm^−2^). The generated ^1^O_2_ was indirectly determined by monitoring the absorbance of ABDA at 410 nm.

The DCFH-DA assay was applied to detect the ROS generation of the DR-CoMo-LDH nanosheets in the presence of H_2_O_2_ (CDT) or under 1567 nm laser irradiation (PDT). For CDT, the DR-CoMo-LDH nanosheets (200 μL, 1 mg mL^−1^) were added in the solution (1800 μL) containing DCFH-DA (6 mM) and H_2_O_2_ (100 μM), and then incubated for 6 min at 25 °C. The generated ·OH was indirectly detected by monitoring the fluorescence intensity at 525 nm. For PDT, the DR-CoMo-LDH nanosheets (200 μL, 1 mg mL^−1^) and DCFH-DA (400 μL, 30 mM) were added into 1400 μL H_2_O and then exposed to 1567 nm laser irradiation for 6 min (0.5 W cm^−2^). The generated ROS was determined by monitoring the fluorescence signal at 525 nm.

### Relative ^1^O_2_ quantum yield measurement

The ^1^O_2_ quantum yield of DR-CoMo-LDH was assessed by SOSG assay and calculated using Rose Bengal as standard (Ф_RB_ = 0.75 in water). 400 μL 30 mM SOSG was added to 1600 μL DR-CoMo-LDH (125 μg mL^−1^) and RB (0.71 μg mL^−1^) solution, respectively. 1567 nm laser (0.5 W cm^−2^) and 550 nm Xenon lamp laser (0.5 W cm^−2^) were employed as the irradiation source, respectively. Fluorescence intensity at 526 nm was measured and recorded after 6 min of irradiation. The relative ^1^O_2_ quantum yield of DR-CoMo-LDH was calculated according to the following formula:

Ф_DR-CoMo-LDH_ = Ф_RB_*A_DR-CoMo-LDH_*I_RB_/A_RB_*I_DR-CoMo-LDH_

A_DR-CoMo-LDH_ and A_RB_ represent the fluorescence intensity of SOSG at 526 nm after adding DR-CoMo-LDH and RB with 1567 nm and 550 nm laser irradiation, respectively. I_DR-CoMo-LDH_ and I_RB_ represent the absorbance of DR-CoMo-LDH and RB at 1567 nm and 547 nm, respectively. Ф_RB_ represents the ^1^O_2_ quantum yield of RB (0.75).

### O_2_ generation assessment

1.8 mL of DR-CoMo-LDH (110 μg mL^−1^) PBS solution (pH = 7.4 and 6.5) was mixed with 200 μL H_2_O_2_ (10 mmol L^−1^). Then, the O_2_ generation was measured by a portable dissolved oxygen meter within 600 s.

### Cell culture

Three cell lines including 4T1, MREpiC and Cos-7 cells were purchased from the Institute of Basic Medical Sciences Chinese Academy of Medical Sciences (Beijing, China). Institute of Basic Medical Sciences Chinese Academy of Medical Sciences used morphology, karyotyping, and PCR-based approaches to confirm the identity of cell lines and to rule out both intra- and interspecies contamination. Also, the cell lines were frequently checked by their morphological features. Three cell lines were cultured in the media of DMEM containing FBS (10%) and streptomycin/penicillin (1%). All cells were cultured in a humidified atmosphere of 5% CO_2_ (37 °C) with a 75 cm^2^ cell-culture flask. The cells were separated from the culture flask by introducing 0.25% trypsin (3 mL) and subcultured for subsequent experiments by adding fresh culture medium.

### In vitro MTT assay

4T1 cells were inoculated into 96-well plates (1 × 10^4^ cells per well) for evaluating biocompatibility or cytotoxicity. The cells were first incubated with culture medium for 24 h to ensure the attachment of cells (37 °C, 5% CO_2_). For in vitro biocompatibility assays, the cells were treated with DR-CoMo-LDH-PEG at various concentrations (ranging from 10 to 200 μg mL^−1^). After co-incubation for 24 h, the previous culture mediums were discarded and 4T1 cells were carefully washed three times with PBS, followed by addition of new fresh culture mediums containing MTT (0.5 mg mL^−1^) at 200 μL per well for another 4 h incubation. The absorbance of the reaction product (formazan) was detected at the characteristic peak of 490 nm by a spectrometer. For in vitro cytotoxicity assays, the cells were divided into six groups: DR-CoMo-LDH-PEG, DR-CoMo-LDH-PEG + 1567 nm laser, DR-CoMo-LDH-PEG + 808 nm laser, DR-CoMo-LDH-PEG + 1567 nm laser + H_2_O_2_, DR-CoMo-LDH-PEG + 808 nm laser + pork tissue, DR-CoMo-LDH-PEG + 1567 nm laser + pork tissue. After co-incubation for 24 h, 4T1 cells were carefully rinsed with PBS for three times and cultured with MTT (200 μL, 0.5 mg mL^−1^) in each well for another 4 h. Subsequently, 4T1 cells were irradiated by 1567 nm or 808 nm laser for 6 min (0.5 W cm^−2^). After every 1 min of laser irradiation, the culture plates were wiped with alcohol cotton for 15 s to avoid the potential temperature rise caused by the laser. Corresponding cell survival rates were determined by aforementioned MTT assay.

### Calcein-AM/PI staining

In order to visualize the MTT results, Calcein-AM/PI staining reagents were adopted to stain live/dead cells based on the green fluorescence imaging of Calcein-AM and red fluorescence imaging of PI. 4T1 cells (a seeding density of 1 × 10^5^ cells per well) were incubated in 6-well culture plates with 2 mL DMEM medium containing 50 μg mL^−1^ of DR-CoMo-LDH-PEG with/without 1567 nm laser irradiation (0.5 W cm^−2^, 6 min) for 24 h, respectively. Then, Calcein-AM (10 μg mL^−1^) and PI (15 μg mL^−1^) mixture was added after the removal of the medium to stain 4T1 cells for 0.5 h. Finally, after discarding the staining solution, 4T1 cells were thoroughly rinsed with PBS, and the fluorescent signal was observed with a Leica fluorescence microscope.

### Apoptosis and necrosis assay

4T1 cells were plated into 6-well plates with a seeding density of 1 × 10^5^ cells per well for 24 h (37 °C, 5% CO_2_). Then, new fresh culture medium containing DR-CoMo-LDH-PEG (50 μg mL^−1^) was added after removing the previous medium. After 12 h, the cells were irradiated with/without 1567 nm laser irradiation (0.5 W cm^−2^, 6 min). Afterward, all treated cells were collected after culture for another 12 h, and quantified by flow cytometer using the Annexin V-FITC/PI assay kit.

### Detection of ROS in vitro

The generated ROS was determined by the DCFH-DA and DHE assays. 4T1 cells were inoculated into confocal laser scanning microscopy (CLSM) culture disks (5 × 10^4^ cells mL^−1^) for 24 h incubation. Afterward, the previous culture medium was discarded and all disks were rinsed three times with PBS, followed by the addition of 2 mL DMEM medium containing 50 μg mL^−1^ of DR-CoMo-LDH-PEG with/without 1567 nm laser irradiation (0.5 W cm^−2^, 6 min) for further 6 h incubation. Then, 4T1 cells were stained with non-fluorescent DCFH-DA (2 mL, 10 μg mL^−1^) or DHE (2 mL, 15 μg mL^−1^) for 0.5 h. Finally, all treated cells were carefully rinsed three times with PBS, and the fluorescence photos were captured via CLSM.

### Detection of mitochondrial membrane potential

4T1 cells were inoculated into CLSM culture disks (5 × 10^4^ cells mL^−1^) for 24 h. After different treatments: (1) Control, (2) 1567 nm laser (0.5 W cm^−2^, 6 min), (3) DR-CoMo-LDH-PEG (50 μg mL^−1^), (4) DR-CoMo-LDH-PEG + 1567 nm laser, (5) DR-CoMo-LDH-PEG + H_2_O_2_ (100 μM) + 1567 nm laser, the cells were rinsed with PBS for three times and further stained with 2 mL of JC-1 (10 µg mL^−1^) for 20 min, and then the fluorescence photos were captured by CLSM.

### Lysosomes disruption assay

4T1 cells were plated into CLSM-exclusive culture disks for 24 h incubation. Then, the cells were exposed to the following treatments: (1) Control, (2) 1567 nm laser (0.5 W cm^−2^, 6 min), (3) DR-CoMo-LDH-PEG (50 μg mL^−1^), (4) DR-CoMo-LDH-PEG + 1567 nm laser, (5) DR-CoMo-LDH-PEG + H_2_O_2_ (100 μM) + 1567 nm laser. Before imaging experiments, all cells were stained with 2 mL of AO (5 μM) for 30 min. Finally, the fluorescence photos were captured by CLSM.

### Western blotting

4T1 cells were plated into 6-well plates (1 × 10^5^ cells per well) for 24 h incubation. After different treatments for another 24 h: (1) Control, (2) 1567 nm laser (0.5 W cm^−2^, 6 min), (3) DR-CoMo-LDH-PEG (50 μg mL^−1^), (4) DR-CoMo-LDH-PEG + 1567 nm laser, (5) DR-CoMo-LDH-PEG + H_2_O_2_ (100 μM) + 1567 nm laser, all cells were lysed and collected. Protein concentrations were determined using BCA protein assay kit (PC0020, Solarbio, China). The proteins were resolved by SDS-PAGE, followed by detection with Western blotting using primary antibodies as follows: anti-GAPDH antibody (ab8245, Abcam, UK, 1:10,000 dilution), recombinant anti-Bcl-2 antibody (ab32124, Abcam, UK, 1:1000 dilution), and recombinant anti-Bax antibody (ab32503, Abcam, UK, 1:10,000 dilution). The secondary antibodies were goat antimouse IgG H&L (ab6789, Abcam, UK, 1:10000 dilution) and goat anti-rabbit IgG H&L (ab6721, Abcam, UK, 1:3000 dilution). The blots were monitored by Mini Chemiluminescent/Fluorescent Imaging and Analysis System (MiniChemi 610).

### Animal experiments

Female Balb/c-nude mice aged 4–6 weeks (20–25 g) were acquired from Beijing Vital River Laboratory Animal Technology Co., Ltd. All mice were placed in stainless steel cages with standard conditions (50% relative humidity and 12 h light/dark cycle) at 25 °C. All animal experiments were monitored and approved by the China-Japan Friendship Hospital Animal Research Center. All procedures performed in this experiment were compliant with the guidelines of the Ethics Committee of China-Japan Friendship Hospital. The right hind legs of all mice were subcutaneously transplanted with 4T1 cells (1 × 10^7^ cells suspended in 100 μL of PBS). The mean tumor had to be 80 mm^3^ for subsequent experiments. The maximal tumor burden permitted by the Ethics Committee of China-Japan Friendship Hospital was 1500 mm^3^. In all animal experiments, the maximal tumor burden was not exceeded.

### In vivo pharmacokinetic studies

For pharmacokinetic analysis, mice bearing 4T1 tumors (*n* = 3) were intravenously administrated with 200 μL of DR-CoMo-LDH-PEG (1 mg mL^−1^). At the specified time points (0, 0.5, 1, 2, 4, 6, 8, 12, 24, and 48 h), the blood was collected, and the concentration of Co ions in the blood was monitored by ICP-AES.

### In vivo antitumor therapy

Mice bearing 4T1 tumors were divided into four groups randomly (6 mice in each group): (1) PBS only, (2) 1567 nm laser only, (3) DR-CoMo-LDH-PEG only, and (4) DR-CoMo-LDH-PEG + 1567 nm laser. The tumor-bearing nude mice in group (1) and (2) were treated with of PBS (200 µL), and mice in group (3) and (4) were treated with DR-CoMo-LDH-PEG (1 mg mL^−1^, 200 µL). Mice in group (2) and (4) received 1567 nm laser irradiation for 6 min (0.5 W cm^−2^). After every 1 min of laser irradiation, the tumors were wiped with alcohol cotton for 15 s to avoid the potentially increased temperature. Tumor volume and weight of mice were monitored every 2 days up to 16 days. Tumor volume was obtained according to the following formula: Volume = tumor length × tumor width^2^ × 0.5.

Tumor volume was defined as *V*/*V*_0_ and measured on day *t* and day 0, respectively.

### In vivo biodistribution study

Mice bearing 4T1 tumors were intravenously administrated with DR-CoMo-LDH-PEG (1 mg mL^−1^, 200 µL) and sacrificed at the indicated time points (4, 8, 12, 24 and 48 h). All tissues including heart, liver, spleen, lungs, kidneys and tumors of mice were collected and digested with concentrated nitric acid. Then the Co ion concentration was determined by ICP-AES to obtain the biodistribution of DR-CoMo-LDH-PEG in different organs and tumors.

### Pathological investigation

After 16 days of treatment, mice bearing 4T1 tumors in four groups were sacrificed. Major organs (containing heart, liver, spleen, lungs, kidneys) and tumor tissues were collected with a paraformaldehyde solution (4%) and embedded in paraffin blocks. After H&E staining, the tissue slices were observed using a Leica fluorescence microscope. For Ki-67 staining, tumor slices were treated with anti-Ki-67 polyclonal antibody (GB121141, Servicebio, China, 1:600 dilution) at 4 °C overnight. Similarly, TUNEL staining was conducted using apoptosis detection kit.

### Immunofluorescence staining of hypoxia

The change of hypoxia in tumor microenvironment was studied after 24 h post injection of DR-CoMo-LDH-PEG (200 µL, 1 mg mL^−1^). The tumor slices were stained with primary antibodies (against HIF-1α (GB13031-1, Servicebio, China, 1:200 dilution)) at 4 °C overnight, followed by phalloidin at 25 °C for 1 h. Images were captured via CLSM. The variation of blood vessels was also investigated after treated with DR-CoMo-LDH-PEG. The tumor slices were stained with primary antibodies (rat-antimouse CD31 antibody (GB113151, Servicebio, China, 1:300 dilution)) to mark blood vessels. Subsequently, the slices were stained with rhodamine-conjugated donkey anti-rat secondary antibody (712-025-150, Jackson ImmunoResearch, USA, 1:200 dilution). The final images of tumor slices were obtained via CLSM.

### Statistical analysis

Data statistics and statistical significance calculation was conducted using Origin 9.0. Microsoft Excel 2019 was applied for tumor volume analysis. Image J 1.8.0 was used to analyze Fluorescence images. NanoScope Analysis 1.5 was utilized for AFM data analysis. All results were expressed as mean ± s.d. The statistical significance of all data was analyzed using one-way analysis of ANOVA, and denoted as: *p < 0.05, **p < 0.01, ***p < 0.001. The 3D models (e.g. molecular structures, cellular structures) in Fig. 1 were mainly produced using the open source software blender. The rest were made through powerpoint 2019, which has been purchased by Beijing University of Chemical Technology and can be downloaded from the official website of the school library.

### Reporting summary

Further information on research design is available in the Nature Research Reporting Summary linked to this article.

## Supplementary information

Supplementary Information

Reporting Summary

## Data Availability

The authors declare that the data supporting the findings of this study are available within the article and the Supplementary Information. Source data are provided with this paper.

## Source data

Source Data
