# Supplementary material for: Defect engineering of layered double hydroxide nanosheets as inorganic photosensitizers for NIR-III photodynamic cancer therapy
Source: Nat Commun. 2022 Jun 13;13:3384. doi: 10.1038/s41467-022-31106-9 (PMC9192653; doi:10.1038/s41467-022-31106-9)
Supplement: Supplementary file 1 — Supplementary Information [file 41467_2022_31106_MOESM1_ESM.pdf]

Supporting Information

Defect Engineering of Layered Double Hydroxide Nanosheets as Inorganic Photosensitizers for NIR-III Photodynamic Cancer Therapy

Weicheng Shen,[1] Tingting Hu,[1] Xueyan Liu,[1] Jiajia Zha,[2] Fanqi Meng,[3] Zhikang Wu,[4] Zhuolin Cui,[1] Yu Yang,[1] Hai Li,[4] Qinghua Zhang,[3,5] Lin Gu,[3,5] Ruizheng Liang,*[1] Chaoliang Tan*[2,6,7]

[1] State Key Laboratory of Chemical Resource Engineering, Beijing Advanced Innovation Center for Soft Matter Science and Engineering, Beijing University of Chemical Technology, Beijing 100029, P. R. China

[2] Department of Electrical Engineering, City University of Hong Kong, 83 Tat Chee Avenue, Kowloon, Hong Kong

[3] Beijing National Laboratory for Condensed Matter Physics, Institute of Physics, Chinese Academy of Sciences, Beijing 100190, China

[4] Institute of Advanced Materials (IAM) and Key Laboratory of Flexible Electronics (KLoFE), Nanjing Tech University (NanjingTech), 30 South Puzhu Road, Nanjing 211816, China

[5] School of Physical Sciences, University of Chinese Academy of Sciences, Beijing 100049, China

[6] Center of Super-Diamond and Advanced Films (COSDAF), City University of Hong Kong, Kowloon, Hong Kong

[7] Shenzhen Research Institute, City University of Hong Kong, Shenzhen, 518057 P. R. China

These authors contributed equally: Weicheng Shen, Tingting Hu.

* Corresponding authors: liangruizheng2000@163.com (R. Liang); chaoltan@cityu.edu.hk (C. Tan).

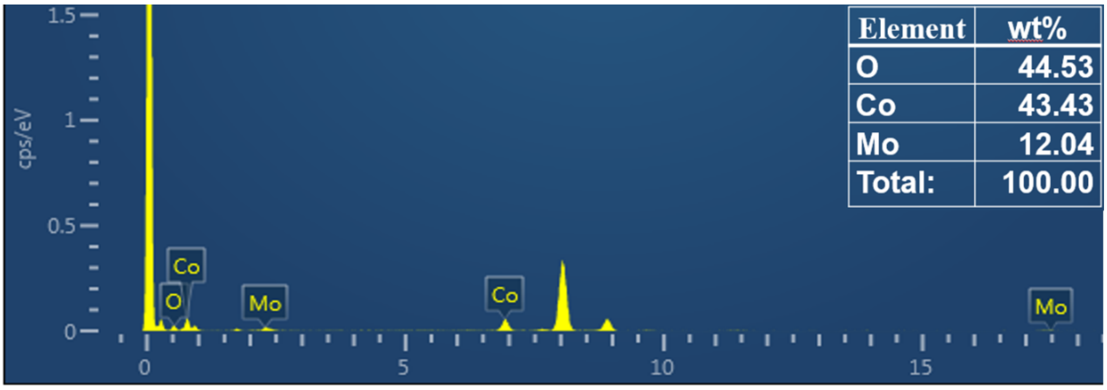

Supplementary Figure 1. EDX characterization of the CoMo-LDH nanosheets. The EDX spectrum of the CoMo-LDH nanosheets. Inset shows the element contents of O, Co and Mo elements.

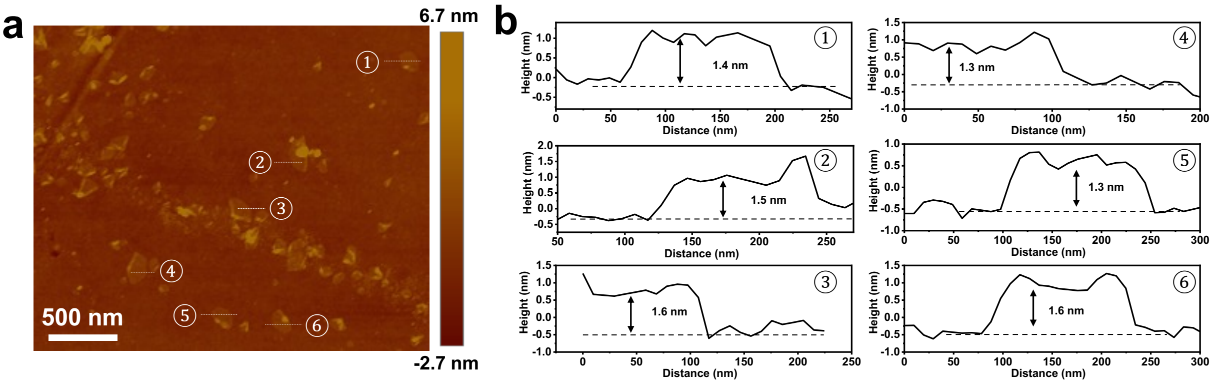

Supplementary Figure 2. AFM characterization of the CoMo-LDH nanosheets. (**a**) The AFM height image of the CoMo-LDH nanosheets and (**b**) its corresponding height profiles measured from 6 nanosheets. Each experiment was repeated three times with similar results.

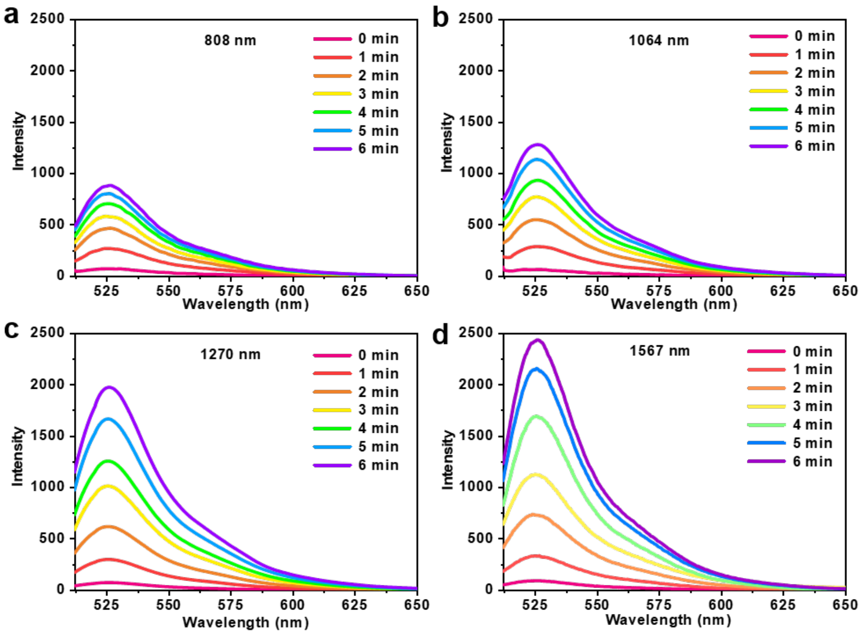

Supplementary Figure 3. Detection of ROS via SOSG assay. The fluorescence spectra of SOSG in presence of the DR-CoMo-LDH nanosheets under different laser irradiation (0.5 W cm^{-2}).

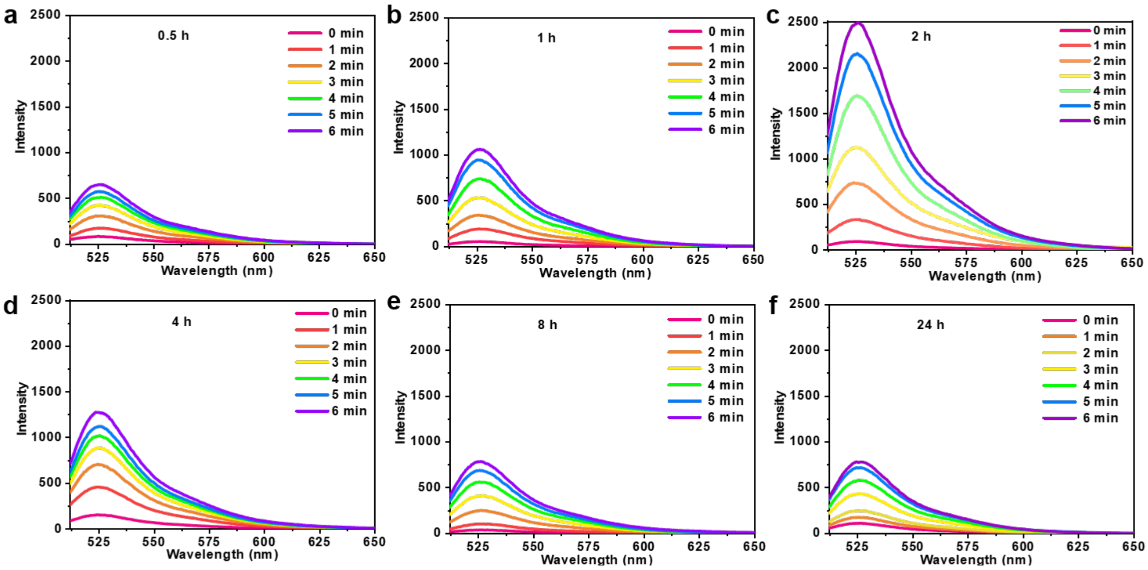

Supplementary Figure 4. Detection of ROS via SOSG assay. The fluorescence spectra of SOSG in presence of the DR-CoMo-LDH nanosheets etched at pH 4.0 for different times under 1567 nm laser irradiation (0.5 W cm^{-2}).

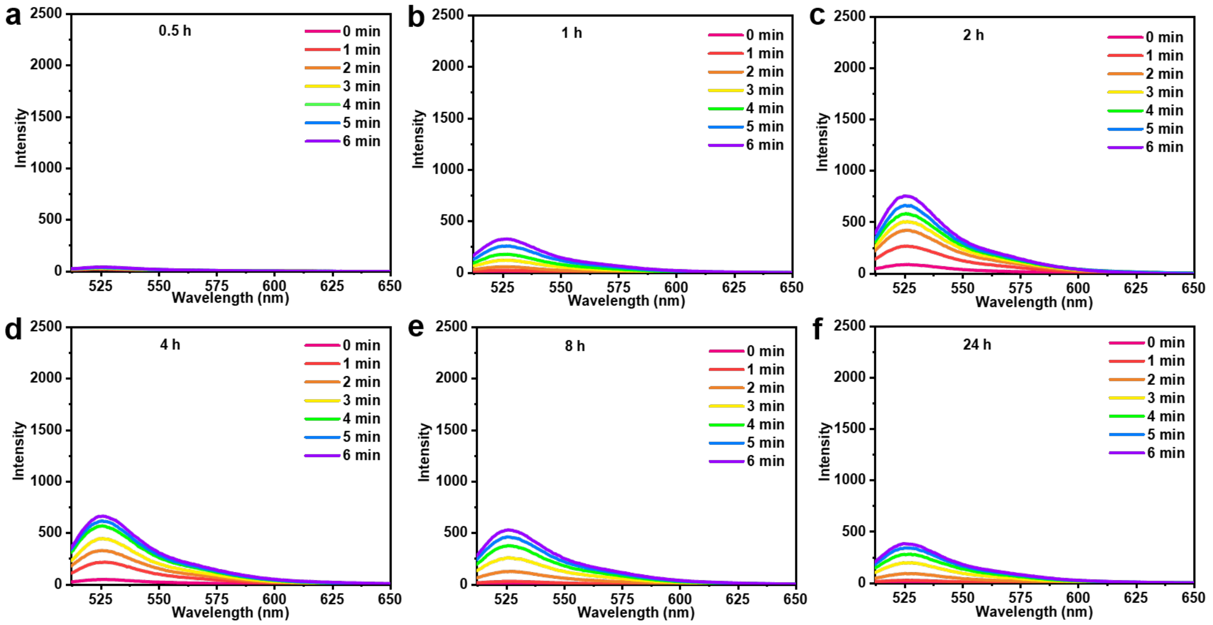

Supplementary Figure 5. Detection of ROS via SOSG assay. The fluorescence spectra of SOSG in presence of the DR-CoMo-LDH nanosheets etched at pH 5.0 for different times under 1567 nm laser irradiation (0.5 W cm^{-2}).

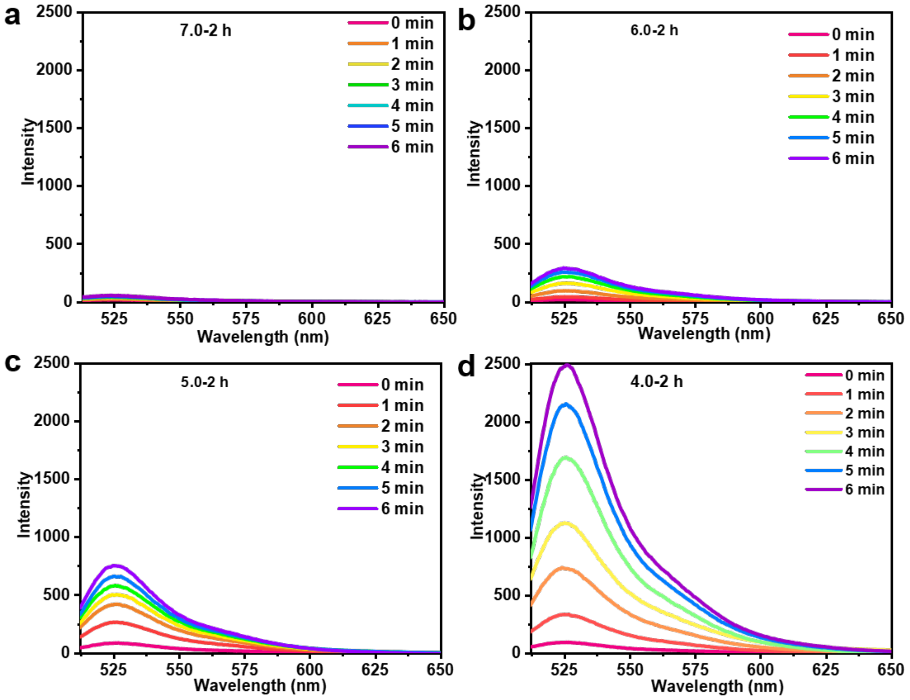

Supplementary Figure 6. Detection of ROS via SOSG assay. The fluorescence spectra of SOSG in presence of the DR-CoMo-LDH nanosheets etched at different pH for 2 h with 1567 nm laser irradiation (0.5 W cm^{-2}).

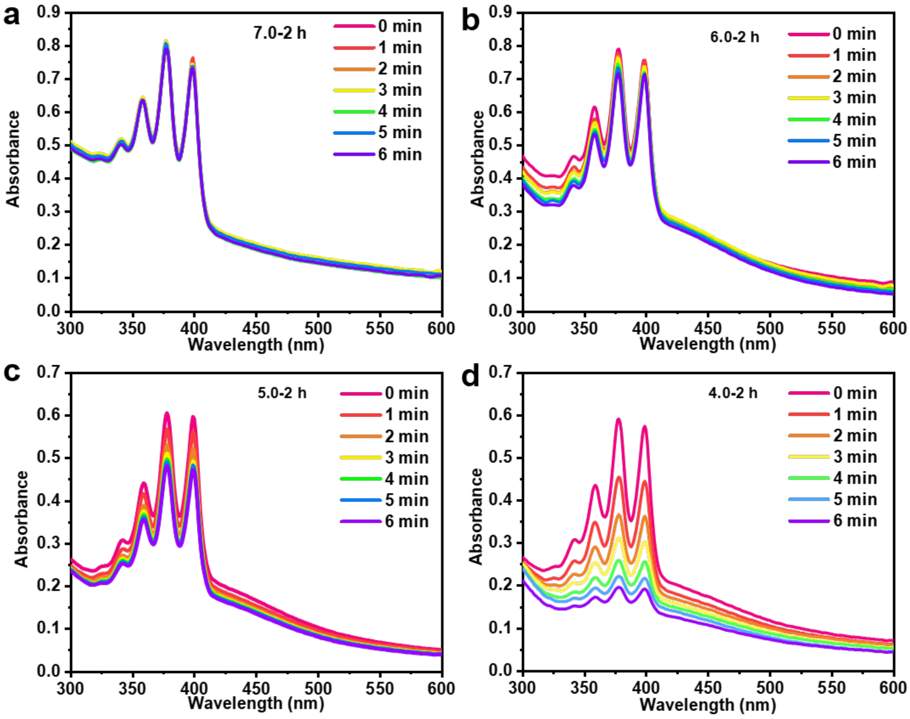

Supplementary Figure 7. Detection of ROS via ABDA assay. UV-vis spectra of ABDA in presence of the DR-CoMo-LDH nanosheets etched at different pH for 2 h under 1567 nm laser irradiation (0.5 W cm^{-2}).

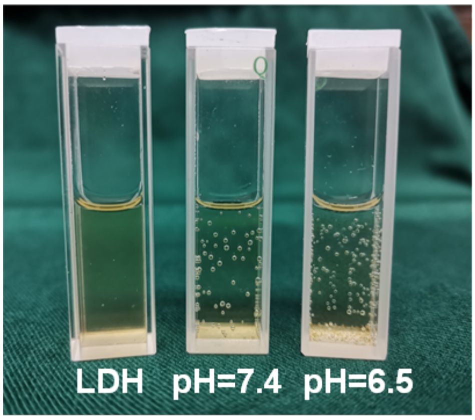

Supplementary Figure 8. O_2 generation assessment of DR-CoMo-LDH nanosheets. O_2 generation photographs of the DR-CoMo-LDH nanosheets without H_2O_2 (left) and with H_2O_2 under pH 7.4 (middle) and 6.5 (right) for 10 min.

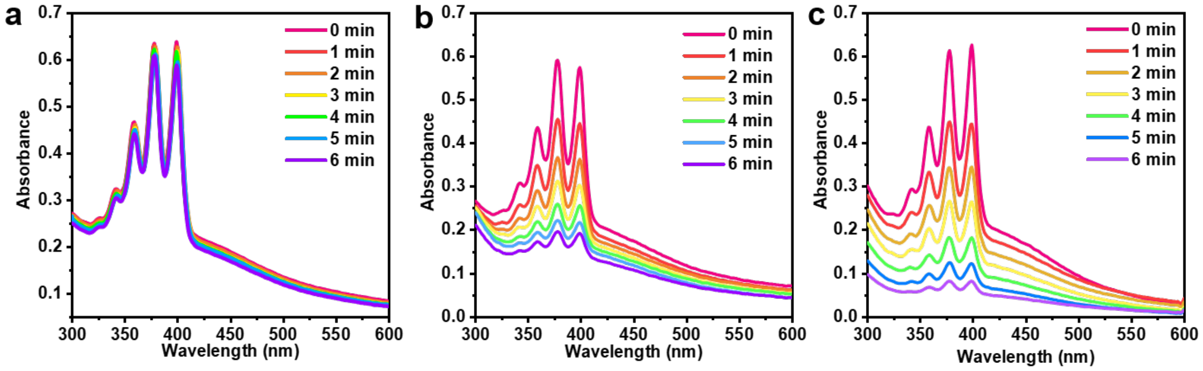

Supplementary Figure 9. Detection of ROS via ABDA assay. UV-vis spectra of ABDA in presence of the DR-CoMo-LDH nanosheets under different environments with 1567 nm laser irradiation (0.5 W cm^{-2}).

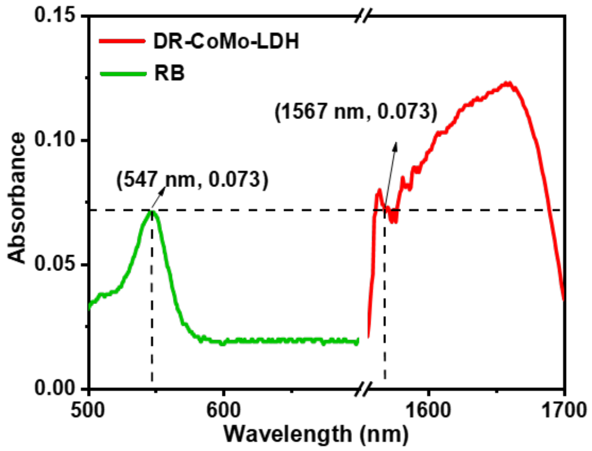

Supplementary Figure 10. UV-vis-NIR diffuse reflection characterization. UV-vis-NIR diffuse reflection spectra of the DR-CoMo-LDH nanosheets and Rose Bengal in aqueous solution.

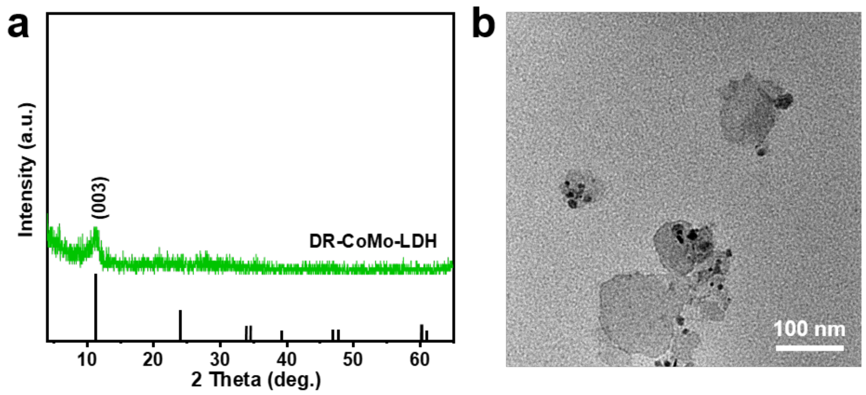

Supplementary Figure 11. XRD and TEM characterization of the DR-CoMo-LDH nanosheets. (**a**) XRD pattern of the DR-CoMo-LDH nanosheets. (**b**) TEM image of DR-CoMo-LDH nanosheets. Each experiment was repeated three times with similar results.

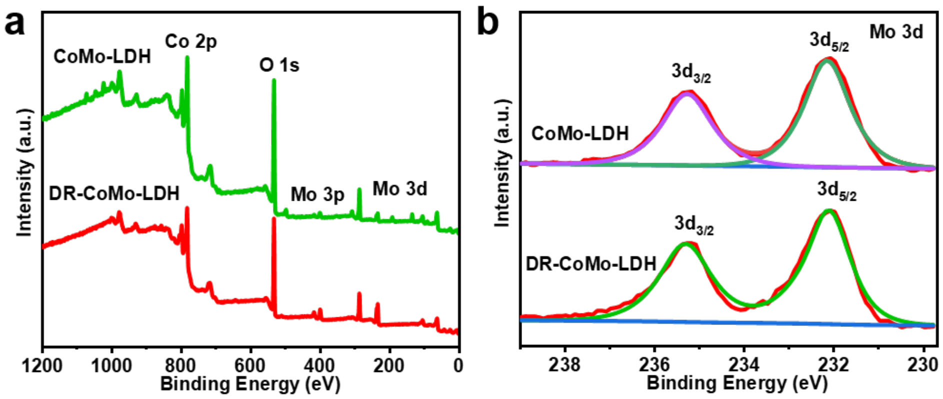

Supplementary Figure 12. XPS characterization of the CoMo-LDH and DR-CoMo-LDH nanosheets. (**a**) XPS spectra of the CoMo-LDH and DR-CoMo-LDH nanosheets. (**b**) Mo 3*d* orbitals of the CoMo-LDH and DR-CoMo-LDH nanosheets.

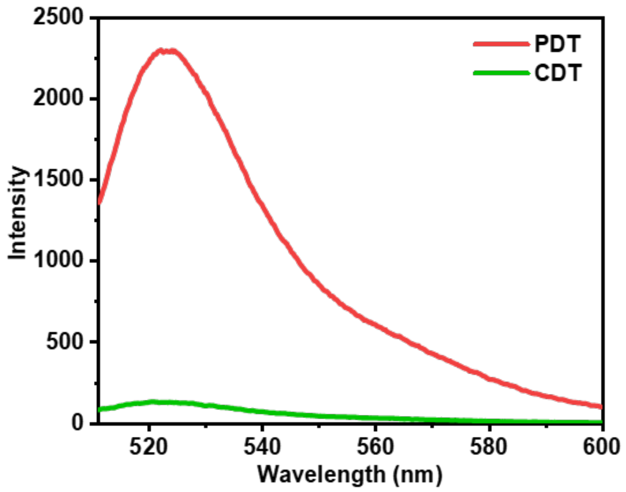

Supplementary Figure 13. Detection of ROS via DCFH-DA assay. Fluorescence spectra of DCFH-DA in presence of the DR-CoMo-LDH nanosheets with adding H_2O_2 (CDT) or under1567 nm laser irradiation (PDT).

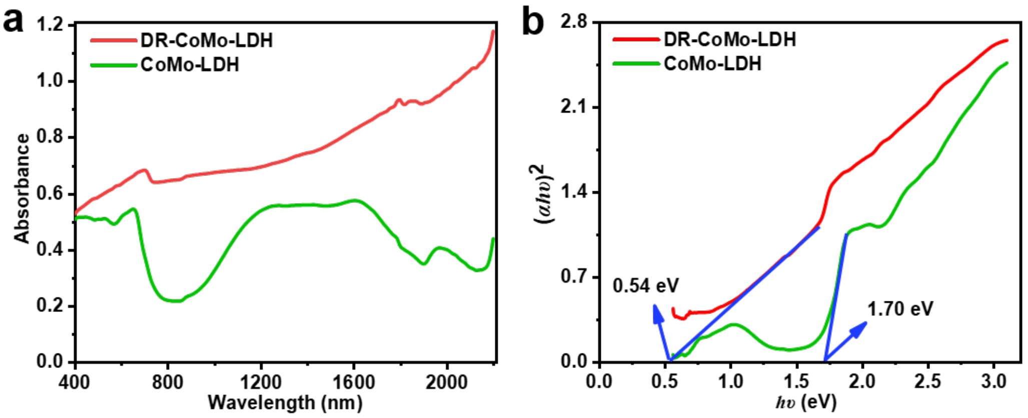

Supplementary Figure 14. UV-vis-NIR diffuse reflection characterization and bandgap extraction. (**a**) UV-vis-NIR diffuse reflection spectrum and (**b**) band gap energy of the CoMo-LDH and DR-CoMo-LDH nanosheets.

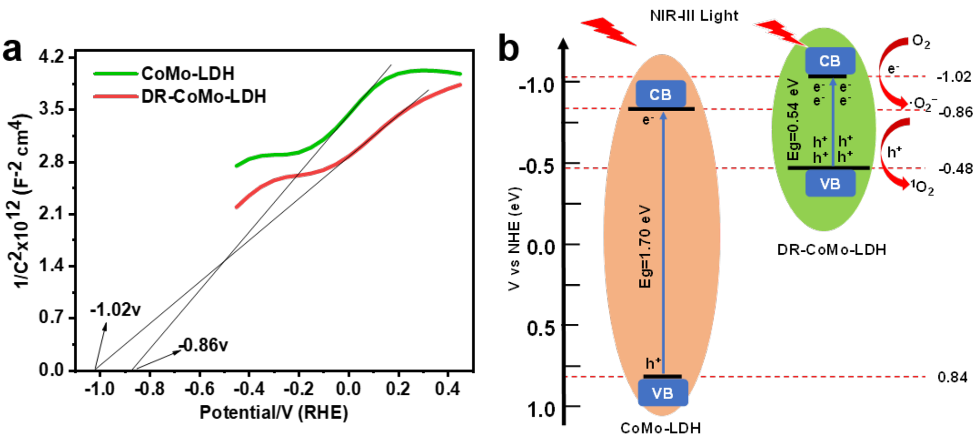

Supplementary Figure 15. **Measurements of bandgaps and band diagrams.** (**a**) Mott-Schottky plots and (**b**) band diagrams of the CoMo-LDH and DR-CoMo-LDH nanosheets.

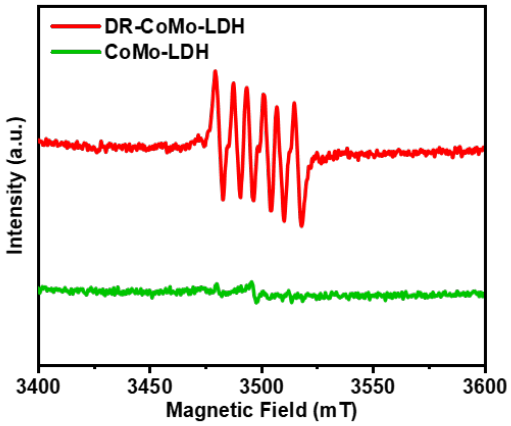

Supplementary Figure 16. **Detection of ROS via ESR spectrometer.** ESR spectra of the CoMo-LDH and DR-CoMo-LDH nanosheets detecting the signal of $\cdot O_2^-$.

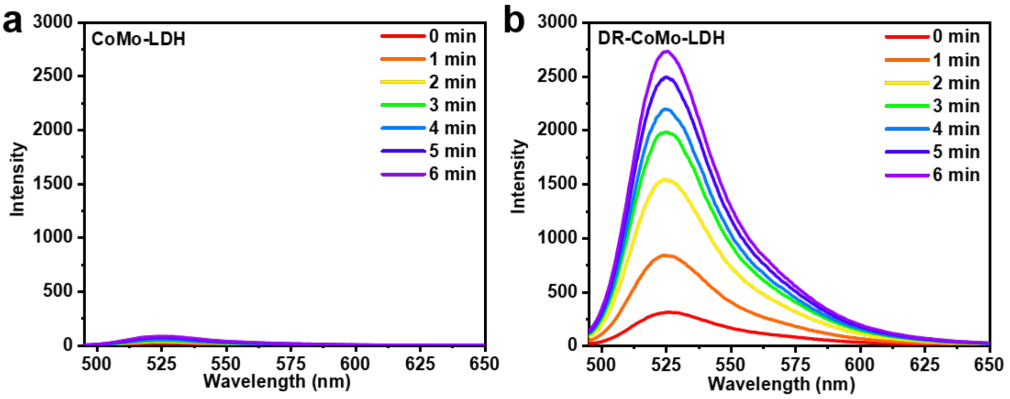

Supplementary Figure 17. **Detection of ROS via DHR123 assay.** DHR123 probe sensing $\cdot O_2^-$ generation in aqueous for (**a**) CoMo-LDH and (**b**) DR-CoMo-LDH under 1567 nm irradiation.

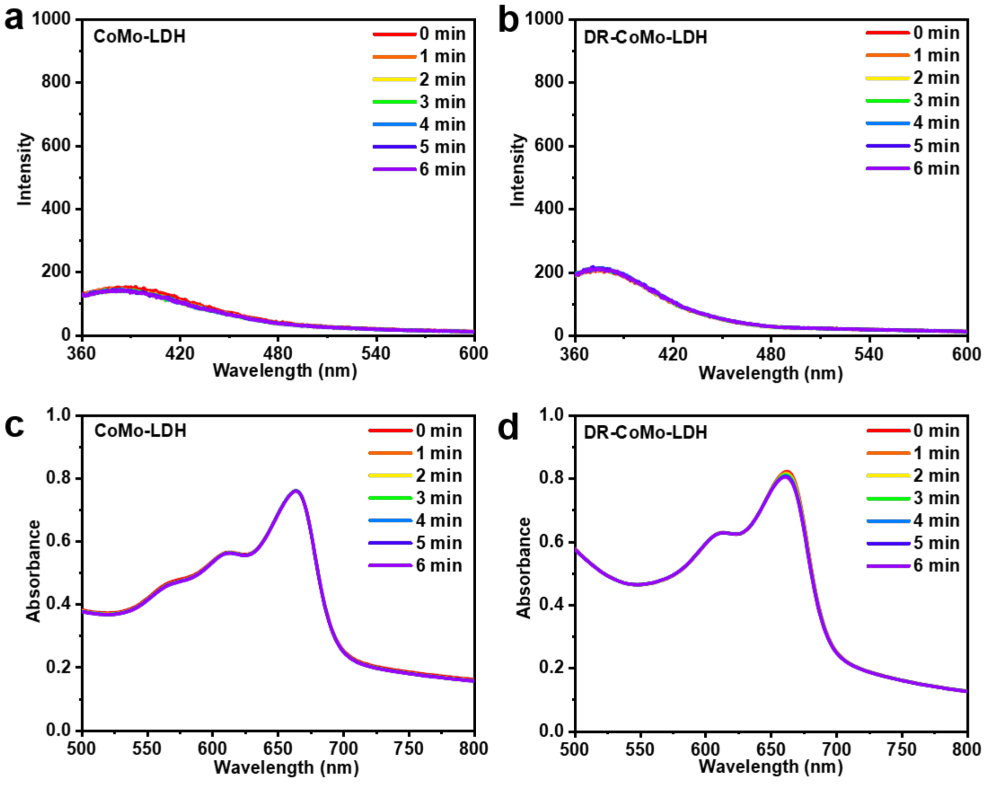

Supplementary Figure 18. Detection of ROS via TA assay. Fluorescence spectra of TA at different time points in the presence of (**a**) CoMo-LDH and (**b**) DR-CoMo-LDH nanosheets under 1567 nm irradiation. The degradation process of MB at different time points treated with (**c**) CoMo-LDH and (**d**) DR-CoMo-LDH nanosheets under 1567 nm irradiation.

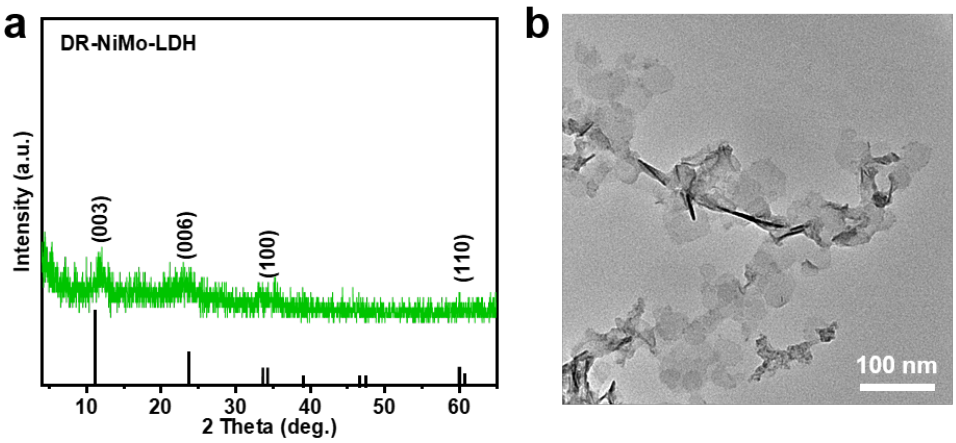

Supplementary Figure 19. XRD and TEM characterization of the DR-NiMo-LDH nanosheets. (**a**) XRD pattern of the DR-NiMo-LDH nanosheets. (**b**) TEM image of DR-NiMo-LDH nanosheets. Each experiment was repeated three times with similar results.

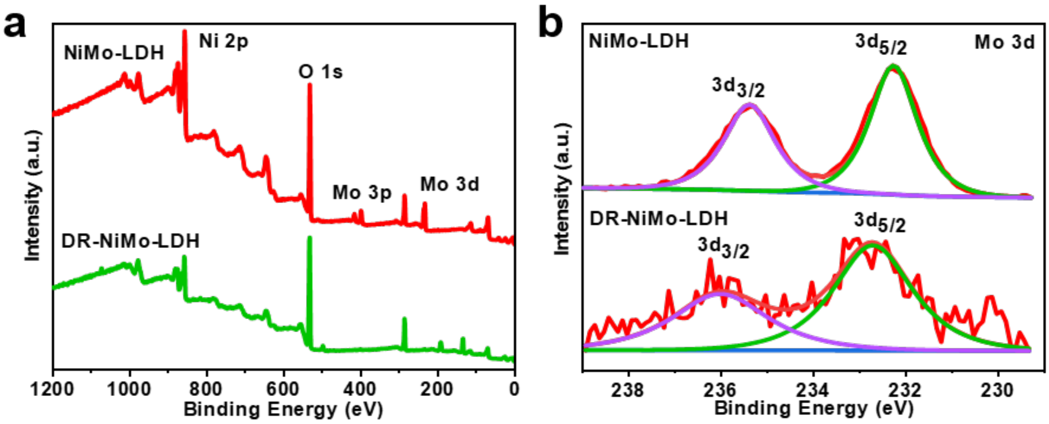

Supplementary Figure 20. XPS characterization of the NiMo-LDH and DR-NiMo-LDH nanosheets. (**a**) XPS spectra of the NiMo-LDH and DR-NiMo-LDH nanosheets. (**b**) XPS Mo 3*d* spectra of the NiMo-LDH and DR-NiMo-LDH nanosheets.

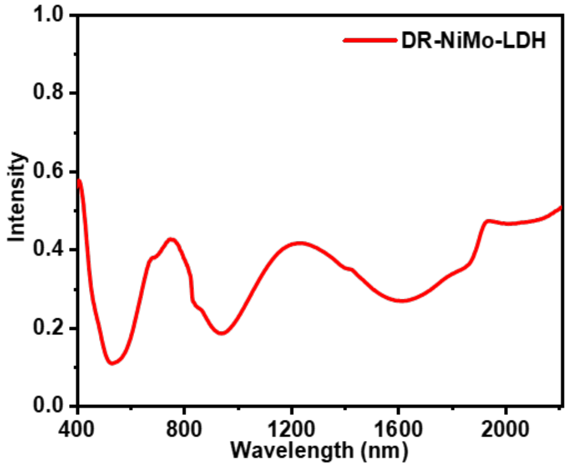

Supplementary Figure 21. UV-vis-NIR diffuse reflection characterization. UV-vis-NIR diffuse reflection spectrum of the DR-NiMo-LDH nanosheets.

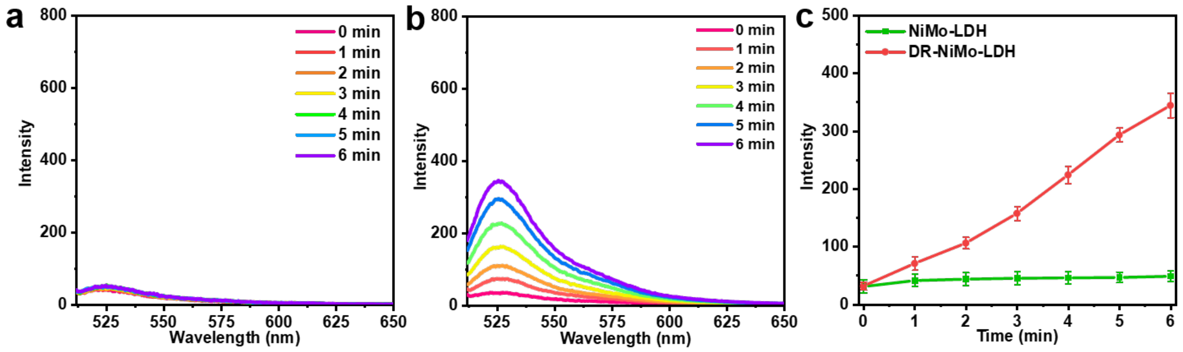

Supplementary Figure 22. Detection of ROS via SOSG assay. The fluorescence spectra of SOSG in presence of (**a**) NiMo-LDH nanosheets and (**b**) DR-NiMo-LDH nanosheets under 1567 nm laser irradiation (0.5 W cm^{-2}). (**c**) The fluorescence intensity of SOSG as a function of irradiation time. Data are presented as mean values ± s.d. (n = 3).

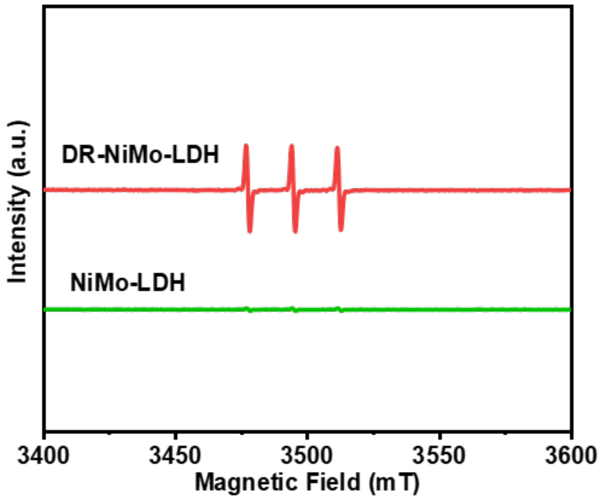

Supplementary Figure 23. Detection of ROS via ESR spectrometer. ESR spectra of TEMP/$^1O_2$ for the NiMo-LDH and DR-NiMo-LDH nanosheets under 1567 nm laser irradiation (0.5 W cm^{-2}, 6 min).

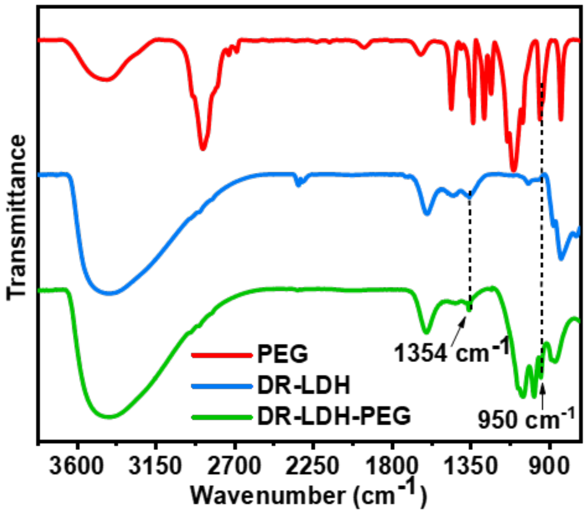

Supplementary Figure 24. FT-IR characterization of the DR-CoMo-LDH and DR-CoMo-LDH-PEG nanosheets. The FT-IR spectra of PEG, DR-CoMo-LDH and DR-CoMo-LDH-PEG.

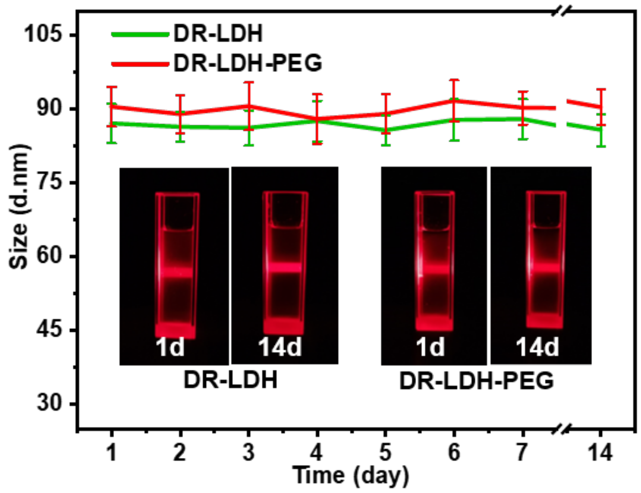

Supplementary Figure 25. DLS characterization of the DR-CoMo-LDH and DR-CoMo-LDH-PEG nanosheets. Stability tests of DR-CoMo-LDH and DR-CoMo-LDH-PEG in water by monitoring their particle sizes for 14 d. Data are presented as mean values ± s.d. (n = 3). (Insets are the digital photographs of DR-CoMo-LDH and DR-CoMo-LDH-PEG dispersed in water for 14 d).

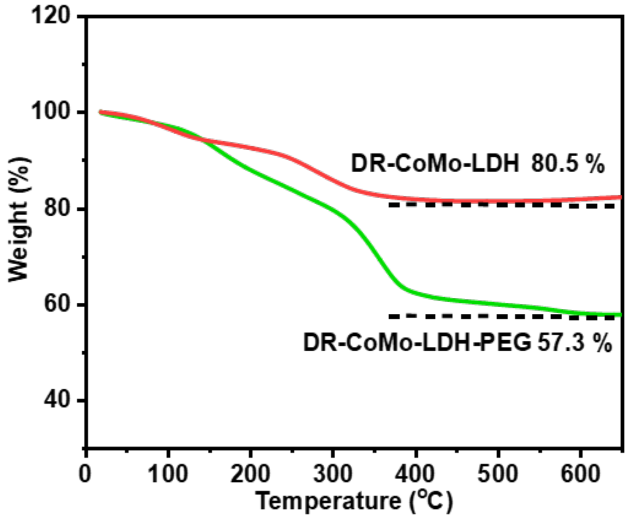

Supplementary Figure 26. TG characterizaiton. TG analysis of DR-CoMo-LDH and DR-CoMo-LDH-PEG nanosheets.

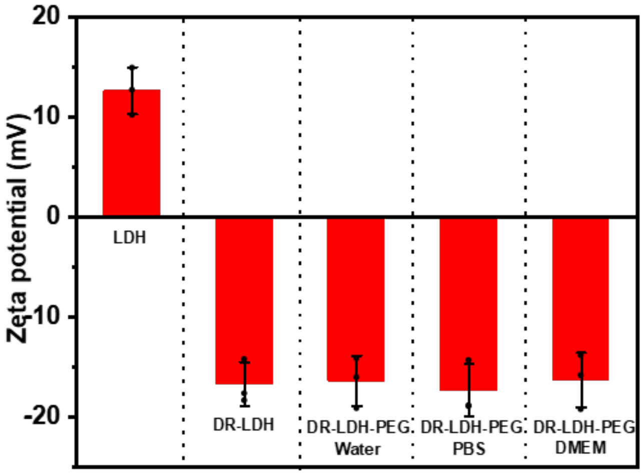

Supplementary Figure 27. Zeta potentials characterization. Zeta potentials of the CoMo-LDH, DR-CoMo-LDH and DR-CoMo-LDH-PEG nanosheets in water, PBS and DMEM. Data are presented as mean values ± s.d. (n = 3).

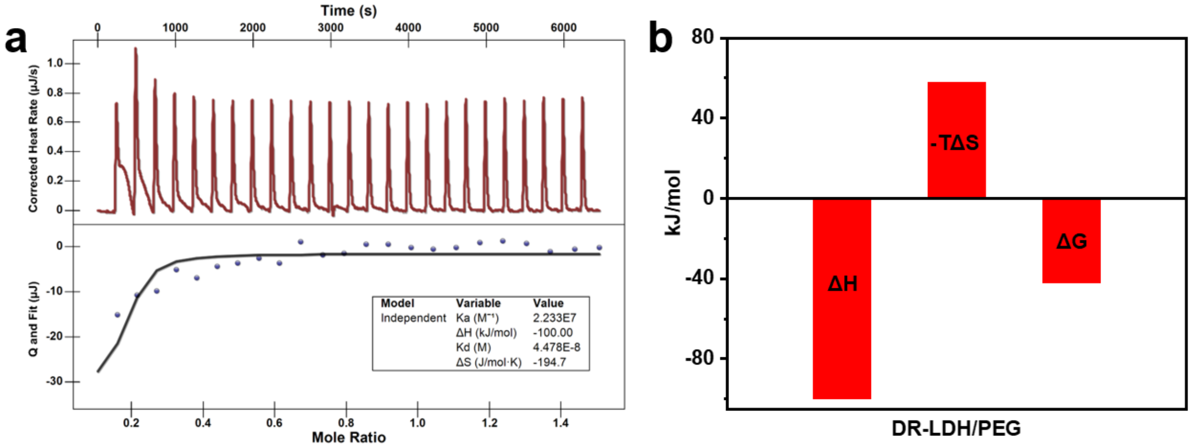

Supplementary Figure 28. Determination of thermodynamic parameters. (**a**) ITC titration measurements of PEG (0.06 mmol L^{-1}) and DR-CoMo-LDH nanosheets (0.3 mmol L^{-1}). (**b**) Thermodynamic parameters for the DR-CoMo-LDH-PEG system.

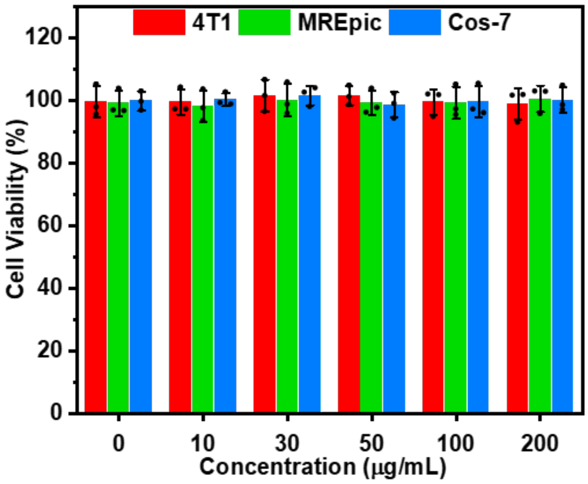

Supplementary Figure 29. In vitro MTT assay. Cell viability of 4T1, MREpiC and Cos-7 cells incubated with various concentrations of DR-CoMo-LDH-PEG. Data are presented as mean values ± s.d. (n = 3).

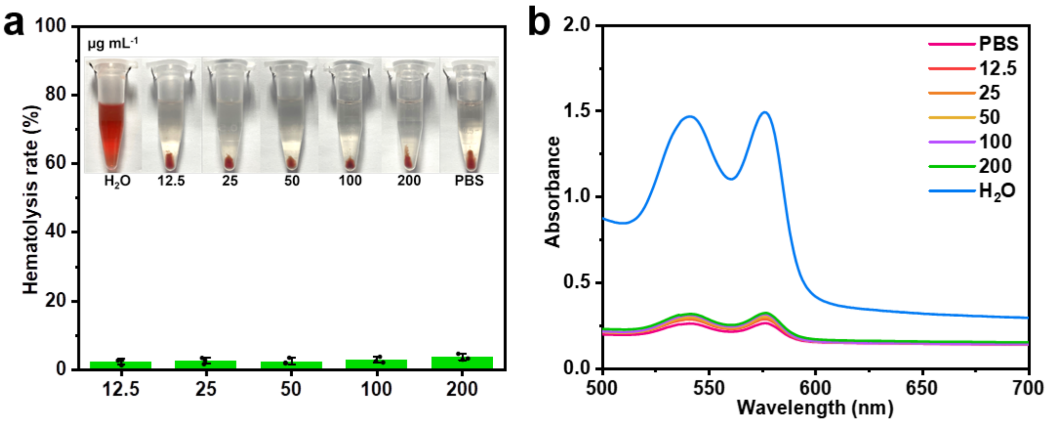

Supplementary Figure 30. Hemolysis assay of DR-CoMo-LDH-PEG. (**a**) Hemolysis rate of the DR-CoMo-LDH-PEG nanosheets with different concentrations toward red blood cells (RBCs) after a 4 h incubation. Inset: the photographs of the samples of H_2O, DR-CoMo-LDH-PEG nanosheets (12.5, 25, 50, 100, 200 μg mL^{-1}), and PBS from left to right. Data are presented as mean values ± s.d. (n = 3). (**b**) The absorption of the RBCs supernatant after being treated by different concentrations of the DR-CoMo-LDH-PEG nanosheets, H_2O and PBS.

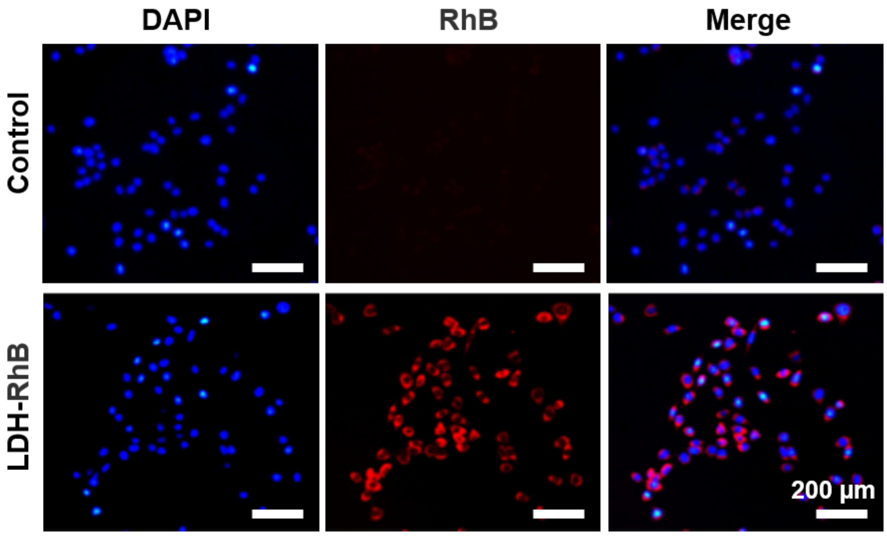

Supplementary Figure 31. Cellular uptake of DR-CoMo-LDH-PEG. Fluorescence images of 4T1 cells treated with the RhB-labeled DR-CoMo-LDH-PEG at 24 h. Each experiment was repeated three times with similar results.

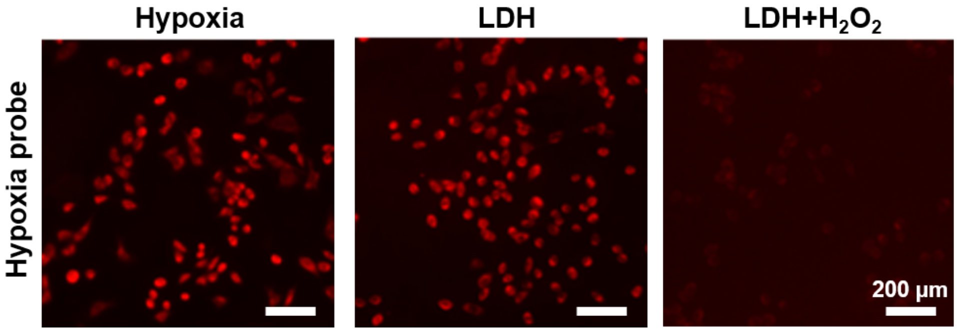

Supplementary Figure 32. Intracellular hypoxia staining assay. Hypoxia level of 4T1 cells detected by red hypoxia detection reagent after different treatments in hypoxic condition. Each experiment was repeated three times with similar results.

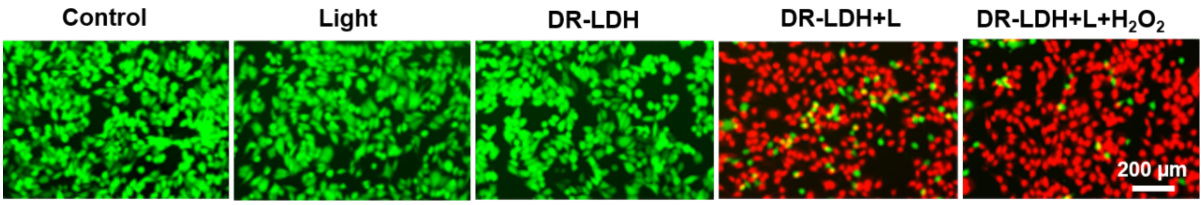

Supplementary Figure 33. Visualization of therapeutic effects via Calcein-AM/PI staining assay. Calcein-AM/PI staining images of 4T1 cells incubated with DR-CoMo-LDH-PEG in different cases: 1) control, 2) 1567 nm laser (0.5 W cm^{-2} for 6 min), 3) DR-CoMo-LDH-PEG, 4) DR-CoMo-LDH-PEG + 1567 nm laser, 5) DR-CoMo-LDH-PEG + 1567 nm laser + H_2O_2 (100 μM). Each experiment was repeated three times with similar results.

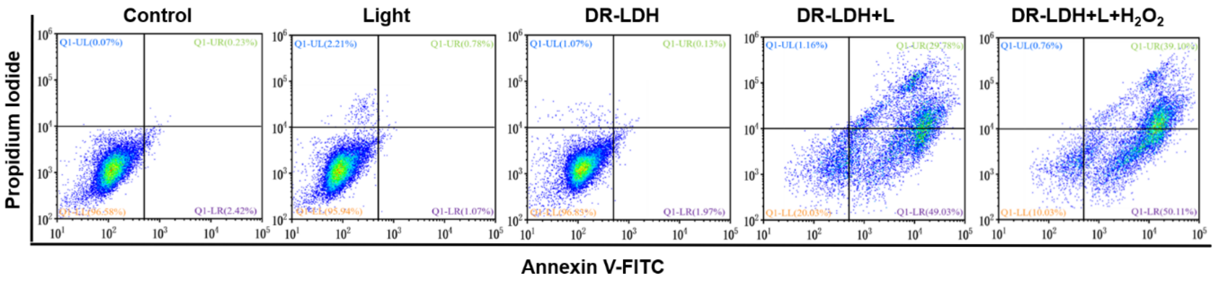

Supplementary Figure 34. Mechanism of cell death induced by DR-CoMo-LDH-PEG. Cell apoptosis analysis using PI/Annexin V-FITC double staining method.

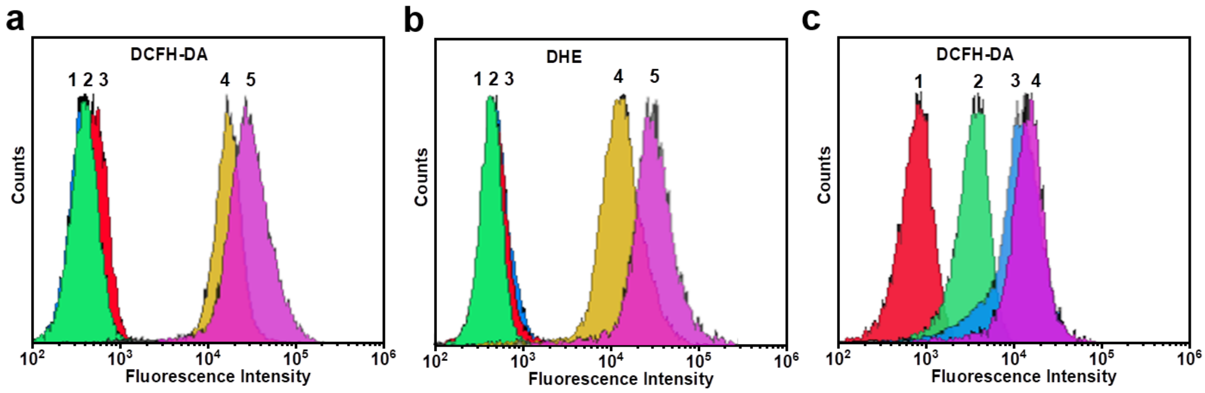

Supplementary Figure 35. ROS quantification via flow cytometer. Flow cytometry quantity of *in vitro* (**a**) ROS and (**b**) $\cdot O_2^-$ production after different treatments: 1) control, 2) 1567 nm laser (0.5 W cm^{-2} for 6 min), 3) DR-CoMo-LDH-PEG, 4) DR-CoMo-LDH-PEG + 1567 nm laser, 5) DR-CoMo-LDH-PEG + 1567 nm laser + H_2O_2 (100 μM). (**c**) Flow cytometry quantity of ROS generation after incubation with DR-CoMo-LDH-PEG under different conditions: 1) 808 nm laser with pork tissue, 2) 808 nm laser without pork tissue, 3) 1567 nm laser with pork tissue, 4) 1567 nm laser without pork tissue.

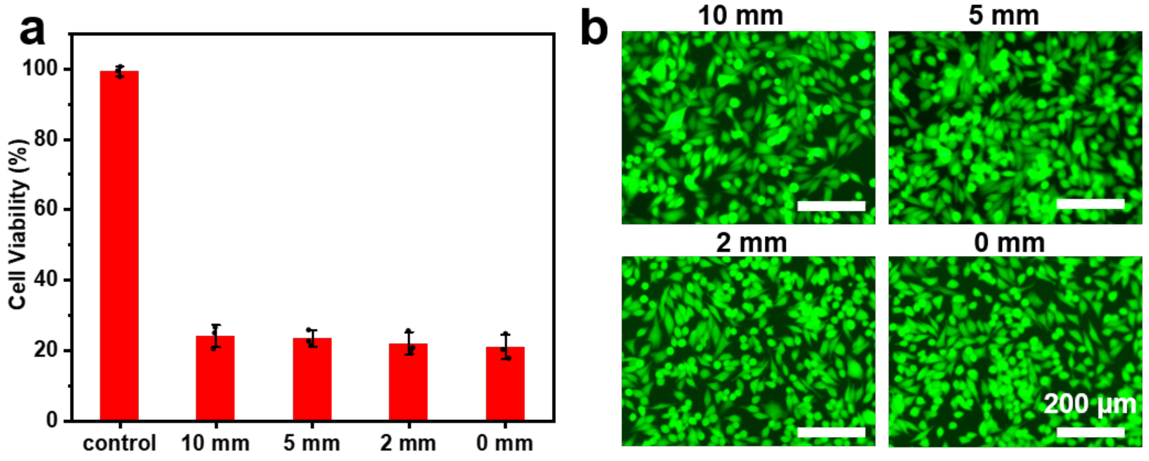

Supplementary Figure 36. In vitro MTT analysis and intracellular ROS staining assay. (**a**) *In vitro* cytotoxicity profiles of 4T1 cells incubated with DR-CoMo-LDH-PEG with or without pork slice under 1567 nm irradiation, and (**b**) corresponding DCFH-DA staining images. Data are presented as mean values ± s.d. (n = 3). Each experiment was repeated three times with similar results.

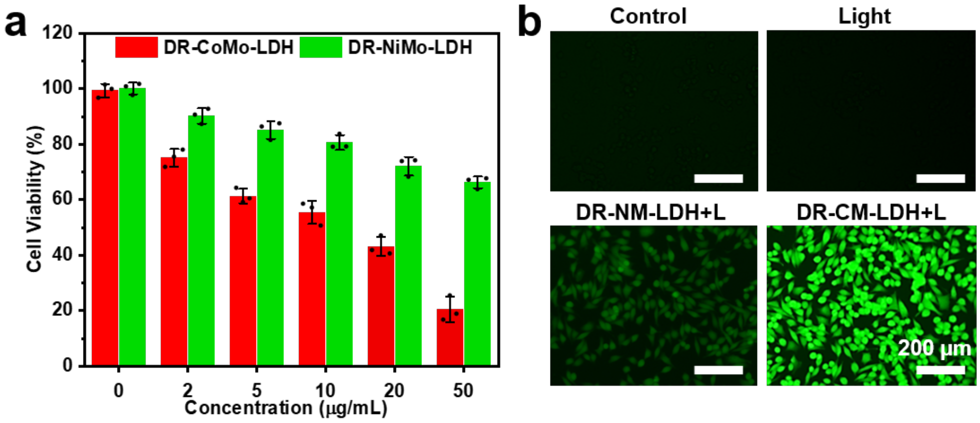

Supplementary Figure 37. **In vitro MTT analysis and intracellular ROS staining assay.** (**a**) *In vitro* cytotoxicity profiles of 4T1 cells incubated with DR-CoMo-LDH-PEG and DR-NiMo-LDH-PEG nanosheets under 1567 nm irradiation. Data are presented as mean values ± s.d. (n = 3). (**b**) DCFH-DA staining images of 4T1 cells in different cases: 1) control, 2) 1567 nm laser (0.5 W cm^{-2} for 6 min), 3) DR-NiMo-LDH-PEG + 1567 nm laser, 4) DR-CoMo-LDH-PEG + 1567 nm laser. Each experiment was repeated three times with similar results.

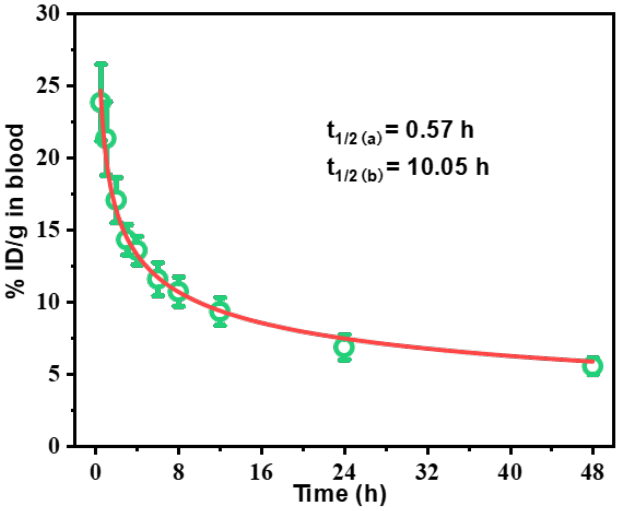

Supplementary Figure 38. Circulation half-life of DR-CoMo-LDH-PEG. Blood circulation time of DR-CoMo-LDH-PEG. The concentration of Co in blood was determined as a function. Data are presented as mean values ± s.d. (n = 3).

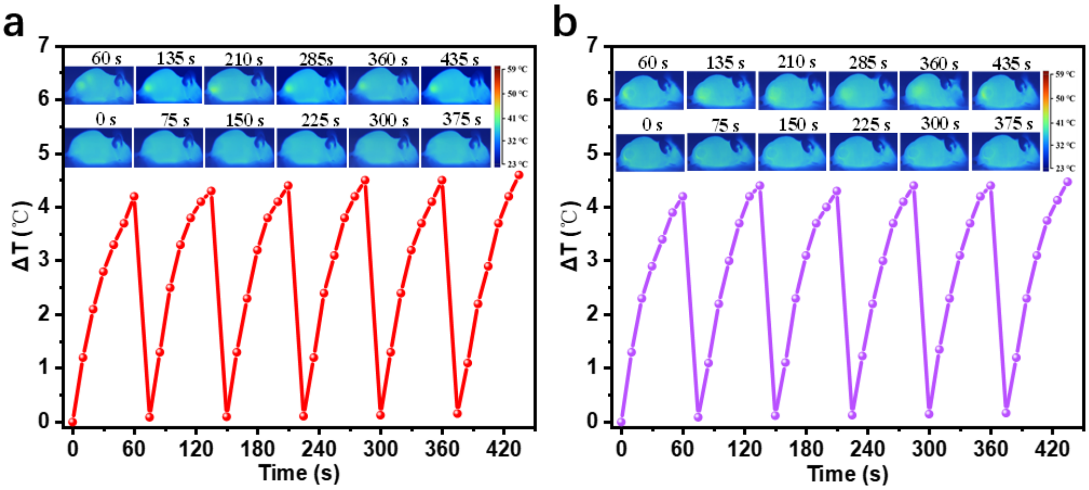

Supplementary Figure 39. Photothermal effect of 1567 nm laser. Temperature variation and photothermal imaging of tumors of mice treated with (**a**) 1567 nm light and (**b**) DR-CoMo-LDH-PEG + 1567 nm light (0.5 W cm^{-2}, 6 min).

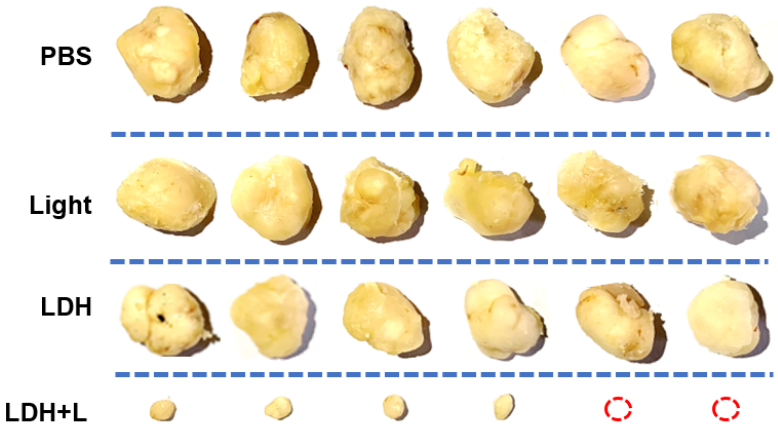

Supplementary Figure 40. The therapeutic effect of DR-CoMo-LDH-PEG. Representative photographs of tumors taken on day 16 after different treatments.

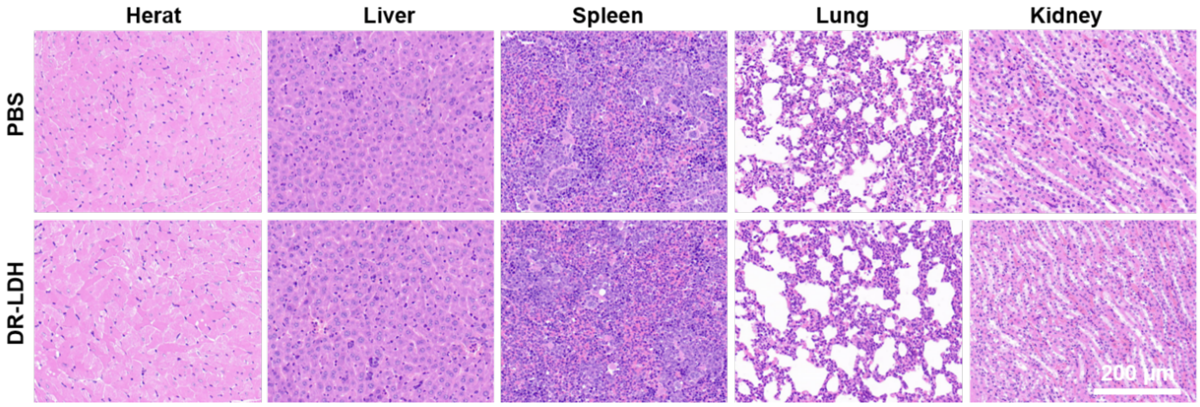

Supplementary Figure 41. Biocompatibility evaluation of DR-CoMo-LDH-PEG via H&E staining assay. Histological images of major organs collected on Day 16 treated with PBS and DR-CoMo-LDH-PEG, respectively. Scale bars are 200 μm. Each experiment was repeated three times with similar results.

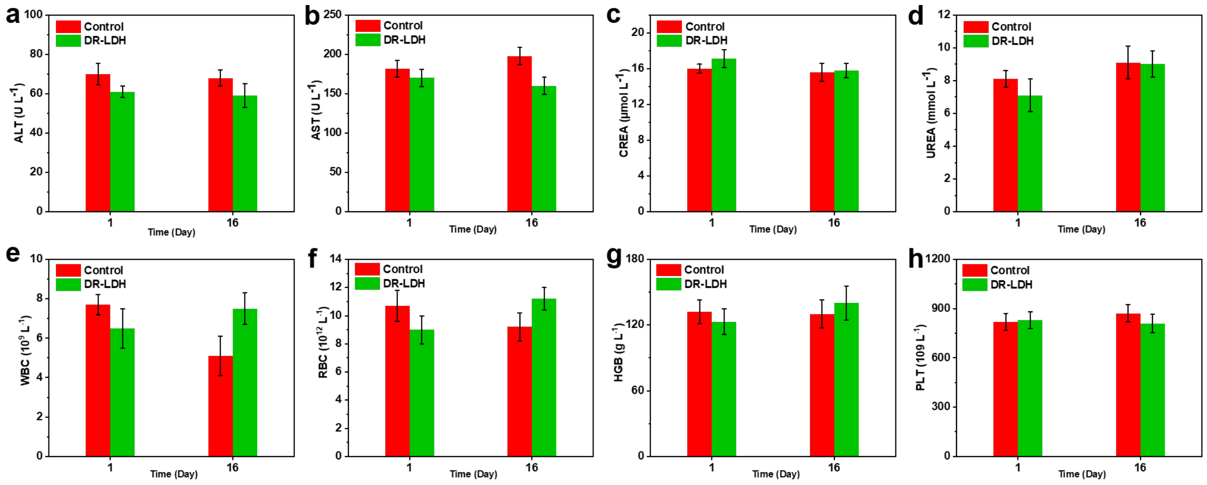

Supplementary Figure 42. Hematology assay of DR-CoMo-LDH-PEG. Kidney and liver function markers and blood cell counts of nude mice bearing 4T1 tumors detected after the injection of saline (control) and DR-CoMo-LDH-PEG at Day 1 and Day 16. Data are presented as mean values ± s.d. (n = 3).

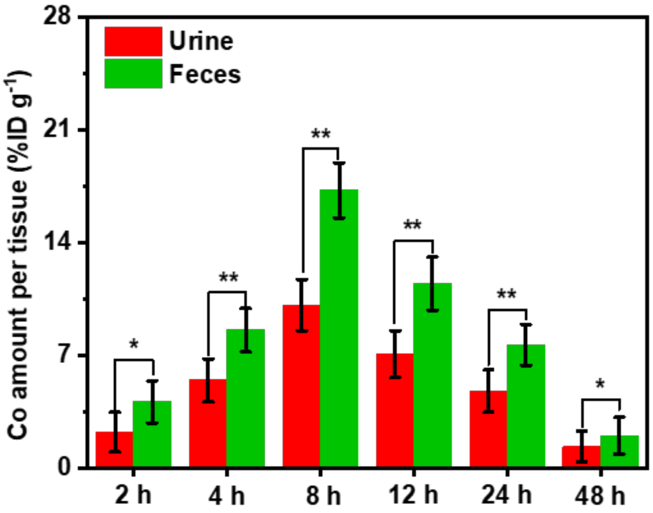

Supplementary Figure 43. Metabolism evaluation of DR-CoMo-LDH-PEG. Excretion of the DR-CoMo-LDH-PEG nanosheets quantified by Co concentration. Data are presented as mean values ± s.d. (n = 3). Statistical analysis was performed via one-way ANOVA. *$p < 0.05$, **$p < 0.01$.
