# Supplementary material for: Defect engineering of layered double hydroxide nanosheets as inorganic photosensitizers for NIR-III photodynamic cancer therapy
Source: Nat Commun. 2022 Jun 13;13:3384. doi: 10.1038/s41467-022-31106-9 (PMC9192653; doi:10.1038/s41467-022-31106-9)
Supplement: Supplementary file 2 — Reporting Summary [file 41467_2022_31106_MOESM2_ESM.pdf]

nature portfolio

Corresponding author(s): Ruizheng Liang, Chaoliang Tan

Last updated by author(s): May 7, 2022

Reporting Summary

Nature Portfolio wishes to improve the reproducibility of the work that we publish. This form provides structure for consistency and transparency in reporting. For further information on Nature Portfolio policies, see our Editorial Policies and the Editorial Policy Checklist.

Statistics

For all statistical analyses, confirm that the following items are present in the figure legend, table legend, main text, or Methods section.

n/a	Confirmed	
☐	☒	The exact sample size (*n*) for each experimental group/condition, given as a discrete number and unit of measurement
☐	☒	A statement on whether measurements were taken from distinct samples or whether the same sample was measured repeatedly
☐	☒	The statistical test(s) used AND whether they are one- or two-sided<br>*Only common tests should be described solely by name; describe more complex techniques in the Methods section.*
☒	☐	A description of all covariates tested
☒	☐	A description of any assumptions or corrections, such as tests of normality and adjustment for multiple comparisons
☐	☒	A full description of the statistical parameters including central tendency (e.g. means) or other basic estimates (e.g. regression coefficient) AND variation (e.g. standard deviation) or associated estimates of uncertainty (e.g. confidence intervals)
☐	☒	For null hypothesis testing, the test statistic (e.g. F, t, r) with confidence intervals, effect sizes, degrees of freedom and P value noted<br>*Give P values as exact values whenever suitable.*
☒	☐	For Bayesian analysis, information on the choice of priors and Markov chain Monte Carlo settings
☒	☐	For hierarchical and complex designs, identification of the appropriate level for tests and full reporting of outcomes
☒	☐	Estimates of effect sizes (e.g. Cohen's d, Pearson's r), indicating how they were calculated

Our web collection on statistics for biologists contains articles on many of the points above.

Software and code

Policy information about availability of computer code

Data collection	TEM data was collected using JEM-2010UHR (JEOL, Tokyo, Japan). XRD data was collected using Shimadzu XRD-6000 diffractometer. AFM data was collected using atomic-force microscope (MultiMode 8, Bruker). ESR spectra data was collected using Bruker EMX1598 spectrometer. STEM data was collected using JEOL ARM200F (JEOL, Tokyo, Japan). XPS data was measured using Escalab 250Xi X-ray photoelectron spectrometer (Thermo Scientific, USA). Hydrodynamic sizes and zeta potentials data were acquired by a Zetasizer UV spectrometer (Malvern Instruments, U.K.). Cellular fluorescence images were performed on a Leica confocal laser scanning microscopy (Leica DM6000M, Germany).
Data analysis	Data statistics and statistical significance calculation was conducted using Origin 9.0. Microsoft Excel 2019 was applied for tumor volume analysis. Image J 1.8.0 was used to analyze Fluorescence images. NanoScope Analysis 1.5 was utilized for AFM data analysis. All results were expressed as mean ± s.d. The statistical significance of all data was analyzed using one-way analysis of ANOVA, and denoted as: *p < 0.05, **p < 0.01, ***p < 0.001.

For manuscripts utilizing custom algorithms or software that are central to the research but not yet described in published literature, software must be made available to editors and reviewers. We strongly encourage code deposition in a community repository (e.g. GitHub). See the Nature Portfolio guidelines for submitting code & software for further information.

Data

Policy information about availability of data

All manuscripts must include a data availability statement. This statement should provide the following information, where applicable:

- Accession codes, unique identifiers, or web links for publicly available datasets
- A description of any restrictions on data availability
- For clinical datasets or third party data, please ensure that the statement adheres to our policy

The authors declare that all data supporting the findings of this study are available within the article and the Supplementary Information. Source data are provided with this paper.

Field-specific reporting

Please select the one below that is the best fit for your research. If you are not sure, read the appropriate sections before making your selection.

[x] Life sciences [ ] Behavioural & social sciences [ ] Ecological, evolutionary & environmental sciences

For a reference copy of the document with all sections, see nature.com/documents/nr-reporting-summary-flat.pdf

Life sciences study design

All studies must disclose on these points even when the disclosure is negative.

Sample size	No statistical methods were used to predetermine the sample sizes. The sample size (generally n≥3 biologically independent samples) was determined by allowable error size and accuracy, and resources. Details regarding sample size of all experiments were provided in the figure captions. For animal experiments, the number of animals in each group was determined by experimental feasibility and sample availability to demonstrate certain results. The size of each sample is in close agreement with those studies already published and with the need for statistical analysis to discuss the degree of differences and measure the variability of these in vivo data.
Data exclusions	No data were excluded from the analyses.
Replication	All the experimental findings were replicated with the number of replicates, animals and variation shown by n and SD. All experimental findings were replicated successfully using biological replicates on different days.
Randomization	Therapeutics study: animals were inoculate with tumor cell suspensions and after randomly distributed into the control and experimental groups. Each specific treatment was administrated to animals according to established schedules and regimens. For other experiments, all samples were randomly allocated into experimental groups.
Blinding	Investigators were blinded when grouping tumor bearing mice, measuring tumor size, performing biodistribution and imaging study. During the experiments designed to evaluate anti-tumor efficacy, animals were inoculated with tumor cells and randomly divided into control and treatment groups. The investigators were blinded during these pre-clinical proof-of-concept studies based on combinatorial schemes. For other experiments, investigators were also blinded to group allocation during data collection and analysis.

Reporting for specific materials, systems and methods

We require information from authors about some types of materials, experimental systems and methods used in many studies. Here, indicate whether each material, system or method listed is relevant to your study. If you are not sure if a list item applies to your research, read the appropriate section before selecting a response.

Materials & experimental systems

n/a	Involved in the study	
[ ]	[x]	Antibodies
[ ]	[x]	Eukaryotic cell lines
[x]	[ ]	Palaeontology and archaeology
[ ]	[x]	Animals and other organisms
[x]	[ ]	Human research participants
[x]	[ ]	Clinical data
[x]	[ ]	Dual use research of concern

Methods

n/a	Involved in the study	
[x]	[ ]	ChIP-seq
[x]	[ ]	Flow cytometry
[x]	[ ]	MRI-based neuroimaging

Antibodies

Antibodies used	anti-Ki-67 polyclonal antibody (GB121141, Servicebio, China), 1:600 dilution<br>against HIF-1α (GB13031-1, Servicebio, China), 1:200 dilution

	rat-antimouse CD31 antibody (GB113151, Servicebio, China), 1:300 dilution<br>rhodamine-conjugated donkey anti-rat secondary antibody (712-025-150, Jackson ImmunoResearch, USA), 1:200 dilution<br>anti-GAPDH antibody (ab8245, Abcam, UK), 1:10000 dilution<br>recombinant Anti-Bcl-2 antibody (ab32124, Abcam, UK), 1:1000 dilution<br>recombinant Anti-Bax antibody (ab32503, Abcam, UK), 1:10000 dilution<br>goat anti-mouse IgG H&L (ab6789, Abcam, UK), 1:10000 dilution<br>goat anti-rabbit IgG H&L (ab6721, Abcam, UK), 1:3000 dilution
Validation	All antibodies were commercially available and were validated by the supplier. All antibodies were used in the study according to the profile of manufacturers. Validation statements are provided on the manufacturer's website.

Eukaryotic cell lines

Policy information about cell lines

Cell line source(s)	4T1, MREpiC and Cos-7 cells were obtained from the Institute of Basic Medical Sciences Chinese Academy of Medical Sciences (Beijing, China).
Authentication	Institute of Basic Medical Sciences Chinese Academy of Medical Sciences used morphology, karyotyping, and PCR based approaches to confirm the identity of human cell lines and to rule out both intra- and interspecies contamination. Also, the cell line were frequently checked by their morphological features.
Mycoplasma contamination	All cells were negative for mycoplasma.
Commonly misidentified lines<br>(See ICLAC register)	No commonly misidentified cell line were used.

Animals and other organisms

Policy information about studies involving animals; ARRIVE guidelines recommended for reporting animal research

Laboratory animals	Female Balb/c-nude mice aged 4–6 weeks (20~25 g) were acquired from Beijing Vital River Laboratory Animal Technology Co., Ltd. All mice were placed in stainless steel cages with standard conditions (50% relative humidity and 12 h light/dark cycle) at 25 ℃.
Wild animals	No wild animals were used in this study.
Field-collected samples	This study did not involve samples collected from the fields.
Ethics oversight	All animals received humane care. All animal experiments were monitored and approved by the China-Japan Friendship Hospital Animal Research Center. All procedures performed in this experiment were compliant with the guidelines of the Ethics Committee of China-Japan Friendship Hospital.

Note that full information on the approval of the study protocol must also be provided in the manuscript.
